# Platelet actin nodules are podosome-like structures dependent on Wiskott–Aldrich syndrome protein and ARP2/3 complex

**DOI:** 10.1038/ncomms8254

**Published:** 2015-06-01

**Authors:** Natalie S. Poulter, Alice Y. Pollitt, Amy Davies, Dessislava Malinova, Gerard B. Nash, Mike J. Hannon, Zoe Pikramenou, Joshua Z. Rappoport, John H. Hartwig, Dylan M. Owen, Adrian J. Thrasher, Stephen P. Watson, Steven G. Thomas

**Affiliations:** 1 Centre for Cardiovascular Sciences, The Medical School, University of Birmingham, Edgbaston, Birmingham B15 2TT, UK; 2 PSIBS doctoral training centre, School of Chemistry, University of Birmingham, Edgbaston, Birmingham B15 2TT, UK; 3 Molecular Immunology Unit, UCL Institute of Child Health, 30 Guilford Street, London WC1N 1EH, UK; 4 School of Chemistry, University of Birmingham, Edgbaston, Birmingham B15 2TT, UK; 5 The Center for Advanced Microscopy and Nikon Imaging Center, Morton 2-681, Northwestern University Feinberg School of Medicine, 303 E. Chicago Avenue, Chicago, Illinois 60611, USA; 6 Brigham and Women's Hospital, Harvard Medical School, Boston, Massachusetts 02115, USA; 7 Randall Division of Cell and Molecular Biophysics, New Hunt's House, King's College London, Guy's Campus, London SE1 1UL, UK

## Abstract

The actin nodule is a novel F-actin structure present in platelets during early spreading. However, only limited detail is known regarding nodule organization and function. Here we use electron microscopy, SIM and dSTORM super-resolution, and live-cell TIRF microscopy to characterize the structural organization and signalling pathways associated with nodule formation. Nodules are composed of up to four actin-rich structures linked together by actin bundles. They are enriched in the adhesion-related proteins talin and vinculin, have a central core of tyrosine phosphorylated proteins and are depleted of integrins at the plasma membrane. Nodule formation is dependent on Wiskott–Aldrich syndrome protein (WASp) and the ARP2/3 complex. WASp^−/−^ mouse blood displays impaired platelet aggregate formation at arteriolar shear rates. We propose actin nodules are platelet podosome-related structures required for platelet–platelet interaction and their absence contributes to the bleeding diathesis of Wiskott–Aldrich syndrome.

## Abstract (web summary)

During early platelet spreading a novel F-actin structure forms, called the actin nodule. Here Poulter *et al.* demonstrate that actin nodule formation depends on WASp and the Arp2/3 complex, and using super-resolution microscopy they show that nodules bear a structural resemblance to podosomes.

## Graphical abstract

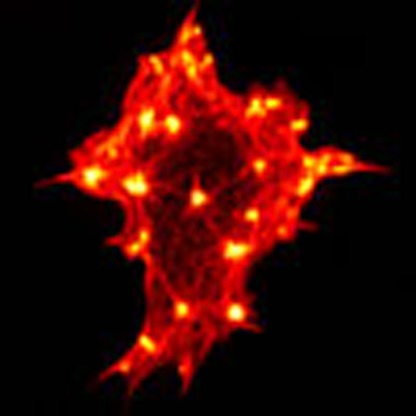

The actin cytoskeleton plays an important role in many processes in platelets and their precursor cell the megakaryocyte^1^,^2^. Actin is essential for the maintenance of platelet morphology and for the rapid change in shape following platelet activation. Actin also regulates platelet surface glycoprotein signalling and thrombus stability^3^,^4^,^5^,^6^,^7^,^8^. In the megakaryocyte, actin plays a critical role in the formation of proplatelets and in mediating degradation of the extracellular matrix (ECM) at sites of podosome formation^9^,^10^,^11^,^12^.

The actin cytoskeleton is assembled into a wide variety of structures including filopodia, lamellipodia, stress-fibres, podosomes, spreading initiation centres (SICs) and actin comets^13^,^14^,^15^,^16^,^17^,^18^. These structures perform various functions and are regulated by a mixture of common and specific pathways. For example, in platelets, severing and uncapping of actin filaments by gelsolin^19^ provides free filament ends for ARP2/3 complex dependent actin polymerization leading to a rapid increase in the F-actin content of platelets^19^. Regulation and organization of ARP2/3-dependent polymerization occurs downstream of members of the Rho GTPases and nucleation promoting factors (NPFs). This is illustrated in platelets by the critical role of Rac1 in lamellipodia formation downstream of both tyrosine kinase and G protein-coupled receptors^4^,^20^. In contrast, the signalling pathway to platelet filopodia formation is unclear with contradictory results from different Cdc42-knockout mouse studies. Pleines *et al.*^21^ used a platelet and megakaryocyte-specific Cdc42 knockout to demonstrate that this GTPase contributed to filopodia formation downstream of the von Willebrand receptor complex, GPIb-IX-V, but was dispensable for filopodia formation on fibrinogen, which binds to integrin αIIbβ3. In contrast, Akbar *et al.*^22^ showed Cdc42 was required for filopodia on fibrinogen and collagen. Furthermore, Rif, another Rho GTPase, which has been shown to be important for generating filopodia in other cells, is dispensable for platelet filopodia formation^23^. Thus, there are cell- and agonist-specific pathways of regulation of actin-based structures.

Recently, we have characterized two specialized actin structures in megakaryocytes and platelets, namely podosomes and nodules, respectively^12^,^24^. Podosomes are actin structures that were originally described in osteoclasts and monocytic cells^25^. They are short-lived, F-actin structures enriched in integrins, Src family kinases (SFK) and the ARP2/3 complex, which are regulated by the activity of Wiskott–Aldrich syndrome protein (WASp). Protein localization is tightly regulated in podosomes and underlies the distinctive structure of an F-actin, ARP2/3 and WASp core surrounded by an integrin, talin and vinculin ring^16^. We have shown that podosomes are required for megakaryocytes to degrade basement membranes enabling proplatelets to reach the bloodstream and form platelets^17^. We speculate that loss of podosome formation may contribute to the reduced count and altered size of platelets in patients with Wiskott–Aldrich syndrome (WAS). However, this alone is unlikely to explain the marked bleeding diathesis in WAS, as individuals with similar platelets counts do not exhibit excessive bleeding.

The actin nodule is a novel actin structure found in platelets during early adhesion and spreading on multiple ECM proteins^24^. Nodules are absent in fully spread platelets where the F-actin is rearranged into stress-fibres. Like podosomes, they are F-actin-rich structures that are dependent on actin polymerization and SFK activity. They contain multiple signalling, actin-binding and adhesion proteins, including Fyn, Rac1, ARP2/3 and integrins. The rapid and early formation of nodules and their absence in fully spread platelets led us to previously speculate that they may play a role in lamellipodia and stress-fibre formation^24^. There have been no further studies on their regulation since our original report and we know very little about their structural organization and regulation. Nevertheless, the similar composition of nodules and podosomes suggests that they may be related structures and may therefore share a dependency on WASp.

There are two members of the WASp family in the mammalian genome, N-WASp and WASp. N-WASp has a ubiquitous distribution; WASp is restricted to haematopoietic cells^26^. Patients with function-disrupting mutations or absence of WASp suffer from WAS and display a range of immune cell related phenotypes, microthrombocytopenia and excessive bleeding^27^. WASp is required for formation of podosomes in megakaryocytes^9^,^12^ and in humans this is associated with a defect in platelet count, size and morphology (in mice, although platelet count is reduced, size is not altered in the absence of WASp^28^). However, it is likely that additional defects contribute to the bleeding diathesis, which is disproportionate to the reduction in platelet count. Although WASp is not required for filopodia formation, it has been shown to be required for normal spreading on fibrinogen and clot retraction^29^,^30^,^31^,^32^. Thus, it appears that multiple defects contribute to the bleeding diathesis in WAS patients.

The aim of this study is to use state-of-the-art microscopy to characterize the organization of proteins at actin nodules and to determine the role of WASp in their regulation. We use a combination of electron microscopy (EM), structured illumination microscopy (SIM) and direct stochastic optical reconstruction microscopy (dSTORM) super-resolution microscopy, and total internal reflection fluorescence (TIRF) microscopy to monitor nodule formation and organization. Moreover, we demonstrate a key role for WASp in nodule formation in human and mouse platelets and that loss of nodules is associated with impaired platelet adhesion and aggregation at arteriolar shear rates, consistent with a critical role for nodules in adhesive processes. Thus, we provide a possible molecular explanation for the bleeding diathesis observed in WAS and for the role of WASp in platelets, namely in regulating nodule formation.

## Results

### Actin nodules form an interconnected network in platelets

‘Conventional' epifluorescent and confocal microscopy have a physical spatial resolution of ∼250 nm in XY and, at best, 500 nm in Z planes due to the diffraction of light through the optics of the microscope. Recently several techniques have been developed to overcome this diffraction limit allowing spatial separation of fluorophores in the order of 10–100 nm in XY (known collectively as super-resolution, reviewed in ref. 33). To investigate the organization of actin and other proteins at the nodule at nanometre resolution, we used SIM and dSTORM on platelets spread on fibrinogen-coated surfaces.

**Figure 1:**
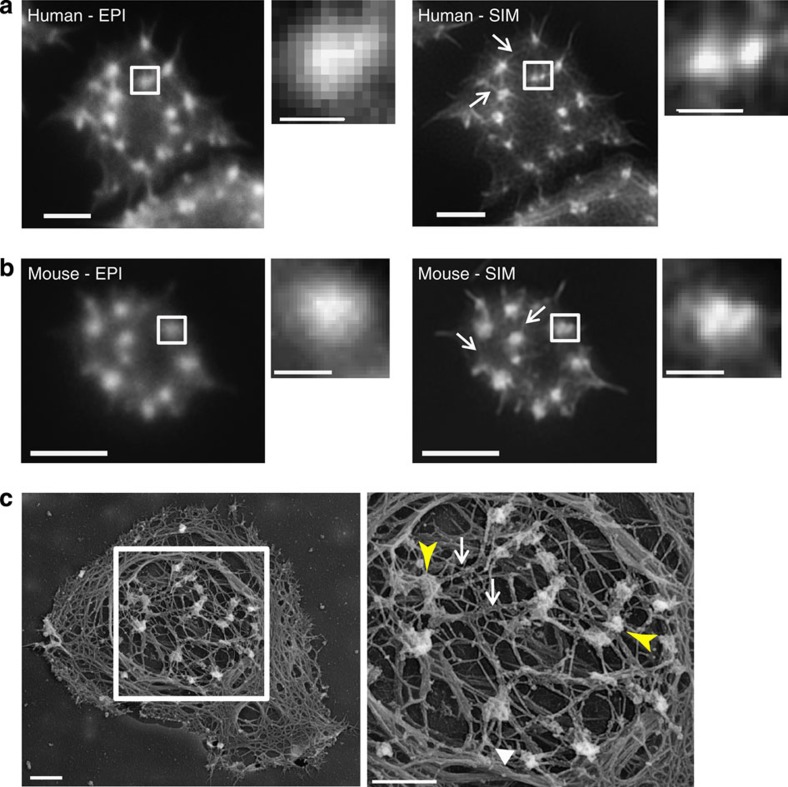
Comparison of SIM and epifluorescent imaging of platelet actin nodules. Epi-fluorescent images (left hand panel) of (**a**) human and (**b**) mouse platelets spreading on fibrinogen and stained for F-actin with Alexa488-phalloidin. The actin nodules in the boxed regions are shown enlarged in the zoomed image. The right hand panel shows SIM images of the same platelets with increased detail seen in the boxed actin nodule shown in the zoomed picture. Arrows indicate the actin fibres radiating from and connecting different actin nodules together. (**c**) Scanning electron micrograph of a representative human platelet displaying actin nodules. The right panel shows an enlargement of the boxed region in the left panel. Regions that could be multiple bright foci of actin are indicated by the arrowheads and interconnecting fibres by the arrows. Scale bars, 2 μm main image, boxed region: 0.5 μm.

The actin cytoskeleton of human platelets was stained with Alexa488-phalloidin and imaged using SIM, which gives an approximate doubling in XY resolution when compared with diffraction-limited epifluorescence (Supplementary Fig. 1a–c). This analysis revealed that actin nodules form part of an interconnected actin network within spreading human platelets (Fig. 1a). Nodules are interconnected via bundles of actin which radiate from their dense actin core (Fig. 1a and Supplementary Fig. 1d, arrows). SIM further reveals that single nodules imaged in diffraction-limited epifluorescence microscopy consist of between 1–4 bright foci of F-actin (Fig. 1a and Supplementary Fig. 1d, arrowheads). This organization of nodules has been observed in platelets from multiple donors in independent experiments. These images are strikingly similar to scanning EM images of platelet cytoskeletons, which show the actin nodules as closely packed F-actin foci linked by actin fibres (Fig. 1c).

We also spread mouse platelets on fibrinogen, stained with Alexa488-phalloidin and imaged using SIM. This again revealed that actin fibres radiate from individual nodules (arrows in Fig. 1b). Interestingly, while some nodules did appear as multiple foci, in general the mouse actin nodules were smaller than those observed in human and were predominantly found as single actin-structures (Compare Fig. 1b with Fig. 1a and Supplementary Fig. 1d).

### Actin nodules link sites of adhesion to the cytoskeleton

To further investigate the structure of nodules, we counterstained human platelets with antibodies against the extracellular portion the αIIb chain of the major platelet integrin αIIbβ3, the actin-binding proteins vinculin and talin or a pan-phosphotyrosine antibody, and analysed them using SIM and dSTORM.

**Figure 2:**
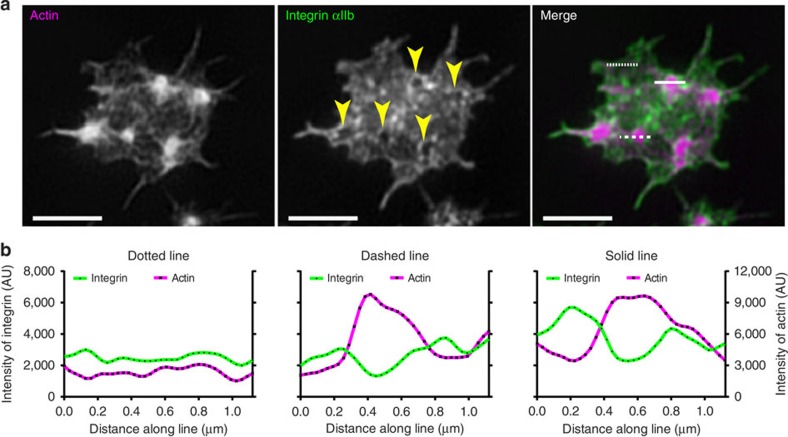
Organization of integrin at actin nodules using SIM. (**a**) Z-projections of a 3D SIM image of a human platelet stained for F-actin with Alexa568-phalloidin (left panel), FITC-anti-αIIb (middle panel) and the merged image (right panel, actin=magenta, integrin=green). Arrowheads in middle panel indicate integrin depleted zones corresponding with the position of actin nodules. (**b**) Line scans showing the intensity of actin (magenta) and integrin (green) fluorescence signal at an area where there is no actin nodule (dotted line), at an actin nodule showing integrin depleted region (dashed line) and at an actin nodule showing integrin depleted region and edge enrichment (solid line). Scale bars, 2 μm.

Using SIM the integrin can be seen to form a diffuse lawn over the platelet surface except at actin nodules where it was either absent from the nodule core or concentrated around the outside of the nodule and absent from the core (Fig. 2a and Supplementary Fig. 2). Line scans of fluorescence intensity for both actin and integrin confirms that in the majority of nodules (∼90% of 774 nodules from 78 cells, *n*=3 experiments) the integrin is depleted from the actin-rich core region (Fig. 2a,b dashed line and Supplementary Fig. 2). Furthermore, ∼50% of platelets also contained some nodules where, in addition to the central depletion, integrin was also enriched at the periphery of the actin core (Fig. 2a,b solid line and Supplementary Fig. 2). These data indicate that either there are subsets of nodules with different patterns of integrin organization or perhaps, more likely, the pattern of integrin distribution at nodules changes over the lifetime of the nodule.

**Figure 3:**
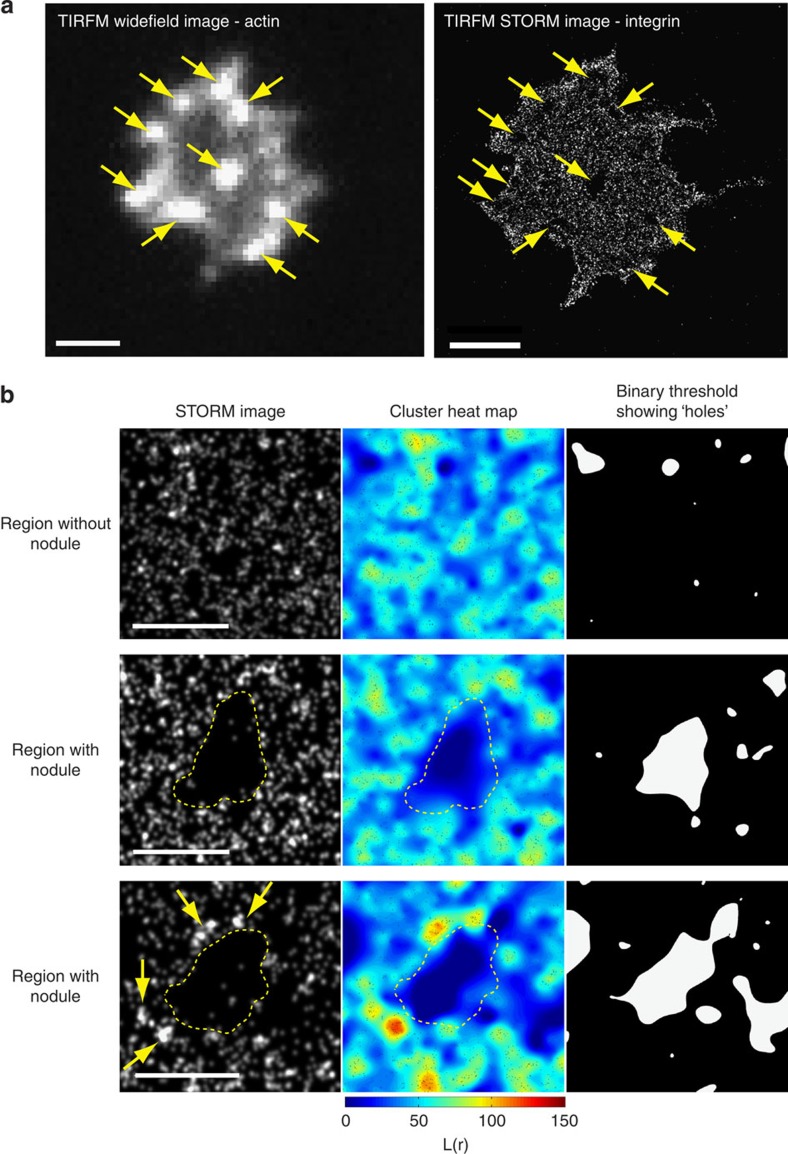
Quantitative analysis of integrin at actin nodules using dSTORM. (**a**) Human platelet stained with Alexa488-phalloidin and imaged for F-actin using diffraction limited TIRF to identify the nodules (left panel) and Alexa647-anti-αIIb and imaged using super-resolution dSTORM TIRF (right panel). Actin nodules and the corresponding integrin depleted zones are indicated by arrows. (**b**) Quantitative cluster mapping analysis of integrin localization on the platelet surface (different cells to that shown in part (**a**)). One micrometre square regions of interest were taken around regions without (top row) and with (middle and bottom rows) actin nodules. For each sample, the left hand panel shows the dSTORM picture, the middle panel shows the cluster heat map and the right hand panel shows the thresholded image showing the integrin depleted zones detected in the distribution of integrin on the platelet surface. The dotted yellow line indicates the position of the F-actin dense core. The actin nodule in the middle row shows no clustering of the integrin around the nodule whereas the one seen in the bottom row appears to show integrin clustering (arrows). Scale bar, **a**: 2 μm, **b**: 0.5 μm.

To further resolve the spatial distribution of integrin at the actin nodule, we used dSTORM in TIRF to monitor αIIb on the platelet surface (Fig. 3). This method is able to localize individual molecules with a precision of ∼20 nm^33^. As before, a lawn of αIIb was observed over the surface of the platelet with clearly defined integrin depleted zones in the centre of the actin nodules (Fig. 3a). Experiments were also performed in which the integrin was labelled after spreading and fixation and these showed the same pattern of integrin staining. This indicated that the depleted region was not due to an unlabelled, internal pool of integrin being brought to the surface during nodule formation. Quantitative cluster mapping, which has been used to analyse the distribution of protein molecules using dSTORM data^34^, identifies molecules displaying a more clustered distribution than would be expected above random. This is presented as a cluster heatmap with ‘hot' colours identifying highly clustered molecules (Fig. 3b) with a high *L*(*r*) value. Analysis of regions of platelets where there are no nodules shows that αIIb is distributed evenly over the surface of the platelet with no significant clustering as indicated by the prevalence of ‘cold' colours in the heatmap (Fig. 3b, top row). Binary threshold analysis of the cluster map indicates very few regions of the platelet where there is no integrin present. In comparison, analysis of the integrin distribution at nodules shows that in all cases an integrin depleted-zone is present at the centre of the nodule (Fig. 3b, Middle row). This is in agreement with SIM data (Fig. 2). In addition, some nodules show regions where integrin molecules appear to be clustered adjacent to the depleted zone indicated by ‘hot' colours in the cluster heat-map (Fig. 3b, bottom row). Together, SIM and dSTORM data indicate that αIIbβ3 is excluded from the platelet surface below the actin nodules and that the pattern of integrin around nodules changes during their lifetime.

**Figure 4:**
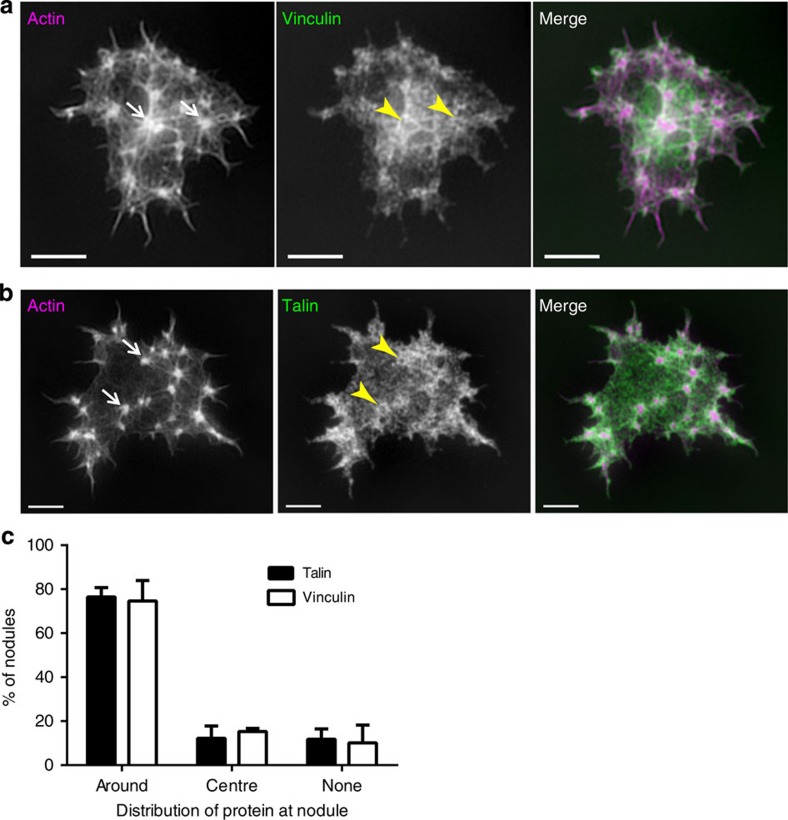
Organization of talin and vinculin at actin nodules using SIM. SIM images of human platelets stained with Alexa488-phalloidin (left panel) and (**a**) Alexa568-anti-vinculin (middle panel) or (**b**) Alexa568-anti-talin. The merged images are shown in the right hand panel (actin=magenta, vinculin/talin=green) showing localization of vinculin and talin at actin nodules. In (**a**) and (**b**), arrows indicate the actin-rich nodule core and arrows head the ring of vinculin or talin around this core. (**c**) Quantification of the pattern of localization of talin and vinculin at the actin nodules by line scan intensity profiles. The line scan profile was scored as either enriched around the nodule; enriched at the centre of the actin nodule core; or no obvious enrichment at the nodule and the percentage of nodules displaying each pattern was plotted. Bars represent mean±s.d. from three independent experiments. In total 738 nodules were measured for talin and 667 nodules were measured for vinculin. Scale bars, 2 μm.

To further these studies, we used SIM to investigate the location of additional actin-binding proteins. We focused on talin and vinculin, which are often found at sites of cell adhesion and are associated with podosomes^16^. SIM revealed that both talin and vinculin are enriched at nodules (Fig. 4a,b), but their distribution is different from that of F-actin. Quantifying the pattern of line scan intensities of actin nodules counterstained with either talin or vinculin into three categories (enriched at nodule periphery; enriched at nodule core; or no obvious enrichment) revealed that 76.4±4.3% and 74.6±9.4% of actin nodules had talin and vinculin, respectively, enriched at their periphery (Fig. 4c; >660 nodules from ≥60 cells, mean±s.d., *n*=3 experiments for each protein). Similar to the results with integrin, a mixture of patterns of localization was observed within individual platelets, which again may reflect kinetic changes during their lifetime.

**Figure 5:**
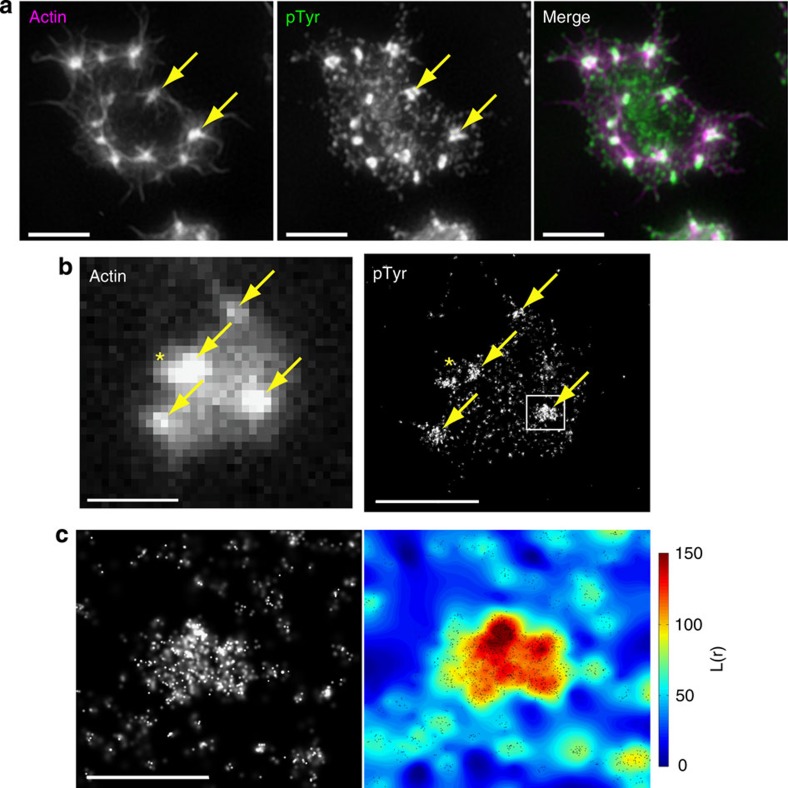
SIM and dSTORM imaging of protein phosphorylation at actin nodules. (**a**) SIM images of a human platelet stained with Alexa488-phalloidin (left panel) and Alexa568-anti-pTyr (middle panel) and the merged image (right panel, actin=magenta, pTyr=green). The presence of tyrosine phosphorylated proteins can be seen as punctate staining across the platelet with a greater intensity and larger foci observed at actin nodules (two examples indicated by arrows). (**b**) TIRF image of Alexa488-phalloidin labelled human platelet (left panel) with arrows indicating the actin nodules and a dSTORM image of the same cell labelled with Alex647-anti-pTyr (right panel). This confirms the results seen with SIM in that tyrosine phosphorylated proteins are more concentrated at actin nodules (arrows). The asterisk indicates an actin nodule that resolves as two separate foci of pTyr labelling in the super-resolution image. (**c**) Enlargement of the boxed region from (**b**) of the pTyr signal at a single actin nodule (left panel). Quantitative cluster mapping of the image (right panel) confirms that phosphorylated proteins are highly clustered at the actin nodule as indicated by the high *L*(*r*) value (according to the pseudocolour scale shown to the right of the cluster map). Scale bars, **a**–**b**: 2 μm, **c**: 0.5 μm.

Protein phosphorylation is important for platelet signalling with many key proteins being phosphorylated during platelet activation. Since nodules are dynamic structures and SFK activity is required for their formation^24^ we investigated whether they were signalling hubs using the presence of phosphotyrosine as a marker of signalling. Both SIM (Fig. 5a) and dSTORM (Fig. 5b) showed that small foci of phosphotyrosine activity are seen across the platelet, and that greater than 90% of actin nodules label strongly with the pan-phosphotyrosine antibody 4G10 (Fig. 5a,b; 546 nodules from 45 cells) indicating that they are sites of signalling activity. Quantitative cluster mapping of 25 nodules and 25 non-nodule regions from dSTORM images indicated that the mean area (±s.d.) of clustered phosphotyrosine labelling at the actin nodule is significantly larger (∼10 times) than clusters seen throughout the remainder of the cell (0.065±0.018 μm^2^ at nodules versus 0.007±0.007 μm^2^ outside of nodules; cluster threshold *L*(*r*)=100, *P*<0.001, Student's *t*-test). Furthermore, there is a significant difference in the molecular density of clustered phosphotyrosine between nodule-associated clusters and the other clusters found throughout the platelet (23,259±10,831 versus 15,130±14,408 fluorescent events detected μm^−2^; *P*=0.008, Student's *t*-test) (Fig. 5c). Taken together, these data indicate that nodules are sites linking interaction with the ECM and the actin cytoskeleton and are sites of tyrosine phosphorylation-mediated signalling activity. These proteins are also enriched at SICs; however, the absence of key SIC markers at nodules indicates that nodules are distinct from SICs (Supplementary Note 1 and Supplementary Fig. 3).

### Actin nodules are dynamic highly regulated structures

The super-resolution data suggest that actin nodules are heterogeneous structures that change their organization over their lifetime. Lifeact-GFP, a 17-amino acid peptide that labels the F-actin cytoskeleton^35^, can be used to monitor formation of F-actin structures without interfering with either their formation or function^36^,^37^. We have carried out several platelet function assays comparing platelets from Lifeact-GFP mice with those from wild-type mice and have found that expression of Lifeact-GFP does not affect their function^37^ and therefore, we used Lifeact-GFP mouse platelets and TIRF to visualize nodule formation at the cell-matrix interface in real time.

**Figure 6:**
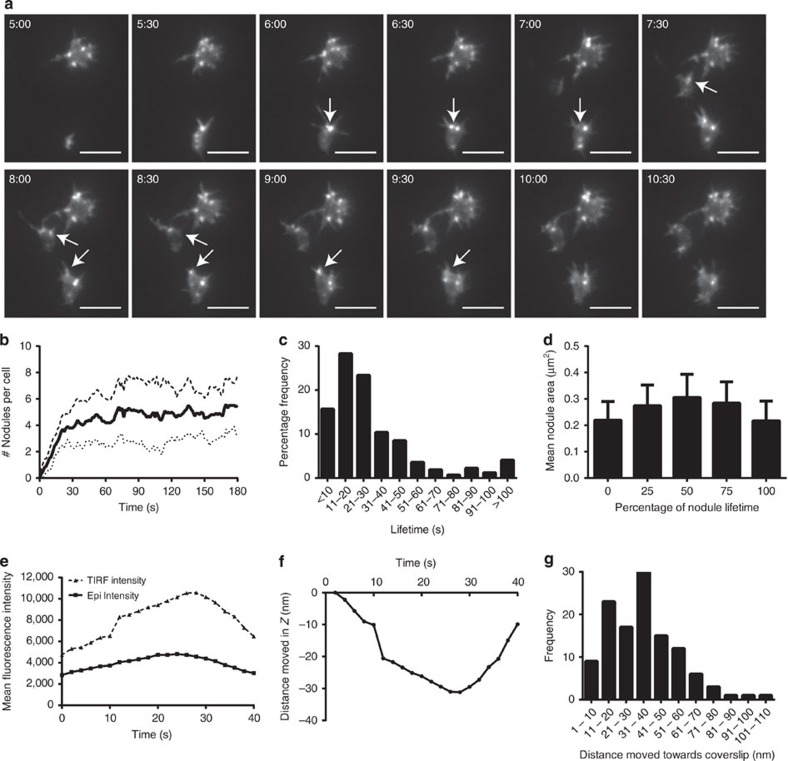
Characterization of actin nodule dynamics by live cell TIRF imaging. (**a**) Frames taken from time-lapse TIRF movies of three representative Lifeact-GFP mouse platelets spreading on fibrinogen (See Supplementary Movie 1) showing the dynamic nature of actin nodule formation and turnover. Arrows indicate turnover of three individual actin nodules. Time stamp=min:s. Scale bar, 5 μm. (**b**) Quantification of the mean number of actin nodules per platelet over time (solid line). Dashed and dotted lines represent±s.d. (**c**) Frequency histogram of the lifetime of individual actin nodules. (**d**) Mean surface area of individual actin nodules normalized for lifetime. Bars represent the mean surface area of the nodule at initial appearance (0% of lifetime), disappearance (100% of lifetime) and at 25, 50 and 75% of their lifetime. Error bars indicate the s.d. For (**b**–**d**) data are means of 20 platelets from two independent experiments. (**e**) Representative mean fluorescence intensity plot of a single actin nodule imaged over time in both TIRF (dashed line) and epifluorescence (solid line). Intensity increases are larger in TIRF than in epifluorescence indicating movement of the nodule in the *z* axis towards the coverslip. This Z movement has been calculated in nm and is shown for this nodule in (**f**). Appearance of the nodule at *T*_0_ is set at 0 nm and movement towards the coverslip is indicated by negative nm values. (**g**) Frequency distribution of movement in Z (in nm) of 118 nodules from three independent experiments.

Time-lapse TIRF movies of Lifeact-GFP platelets settling onto fibrinogen-coated glass-bottomed dishes demonstrated that nodules rapidly form as platelets adhere and begin to spread (Fig. 6a and Supplementary Movie 1). Subsequently, the platelets continue to make nodules and filopodia as the cells spread out to form the characteristic spiky appearance of mouse platelets on fibrinogen. The number of actin nodules per mouse platelet increases rapidly in the first 20 s of spreading and then levels off, averaging between four and six nodules per platelet (Fig. 6b). The average diameter (measured at the widest point) of an actin nodule in fixed samples was 0.50±0.12 μm (mean±s.d.) with the majority (>95%) of them in the range 0.25 to 0.62 μm. The median lifetime of an actin nodule was 22.1 s (25% percentile=13.5 and 75% percentile=36.3;>400 nodules) (Fig. 6c).

We analysed the surface area of the nodules over their lifetime to determine whether they change in their appearance. We synchronized the size measurements by recording surface area in the first and last frame that the nodules were present and then at 25, 50 and 75% of their lifetime (Fig. 6d). These data indicated that nodules are fairly uniform in size at appearance and disappearance (surface area at appearance=0.22±0.07 μm^2^ and at disappearance=0.22±0.07 μm^2^, mean±s.d.); however, over the course of their lifetime they increase in size by up to 50% at mid-lifetime (0.31±0.09 μm^2^). This increase was significant compared with that at appearance and disappearance (*P*<0.001; One way ANOVA & Tukey's multiple comparison). This change may reflect the ‘flattening' down of the nodules onto the surface rather than a change in size. We investigated this further by imaging the same actin nodules in both TIRF and Epifluorescence (Supplementary Movie 2). Increased actin polymerization will result in increased fluorescence intensity in both modalities. However, we observed a consistent increase in TIRF intensity above that of Epifluorescence (Fig. 6e) suggesting that the nodules were increasing in size and also moving downwards in Z, and therefore into the higher intensity region of the TIRF evanescent wave^38^. This movement in Z was quantified by inputting the TIRF intensity values from 118 nodules (*n*=3 experiments) into equation 4 (ref. 39) to generate the mean (±s.d.) distance moved towards the substrate. This was calculated as 34.6±19.0 nm, which is in the order of the size of integrins^40^. These data support the hypothesis that nodules both increase in size, due to actin polymerization, and change their position in Z during their lifetime. We also measured the lateral displacement or mobility of individual actin nodules from TIRF timelapses and found all actin nodules are fixed in their location (Supplementary Fig. 4). Together these data indicate that actin nodules are formed rapidly upon platelet activation/adhesion, are tightly controlled in terms of size and lifetime and are immobile in XY but move towards the substrate in Z. These properties are also observed for megakaryocyte podosomes; however, in contrast to podosomes, we have shown that actin nodules do not degrade the fibrinogen matrix (Supplementary Note 2 & Supplementary Fig. 5).

### WASp is required for actin nodule formation

**Figure 7:**
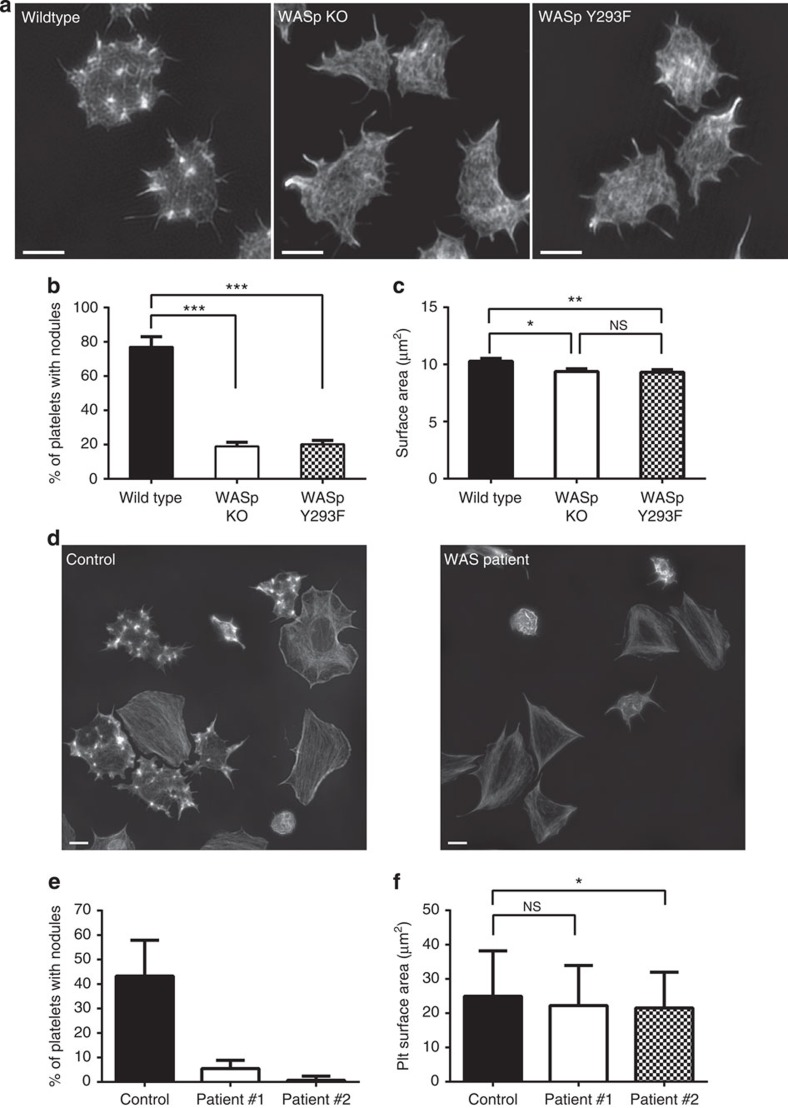
WASp is required for the formation of actin nodules in mouse and human platelets. (**a**) SIM images of Alexa488-phalloidin labelled platelets from wild type (left panel), WASp KO (middle panel) and WASp Y293F (right panel) mouse platelets. Platelets from both the WASp KO and WASp Y293F did not form conventional actin nodules as seen in wild-type platelets. (**b**) Quantification of the number of platelets displaying actin nodules. Data are means±s.d. (*n*=3). ***P<0.001 from one way ANOVA. (**c**) Quantification of the surface area of spread platelets from wild type, WASp-KO and WASp-Y293F mice. Data are means±s.d. (*n*=3), *P<0.05 from one way ANOVA. Approximately 400 platelets were analysed for each genotype. (**d**) SIM images of Alexa488-phalloidin labelled human platelets from control (left panel) and Wiskott–Aldrich syndrome patient (WAS patient#2—right panel). Platelets from the patient samples did not form actin nodules as could be seen in the control samples. (**e**) Quantification of the percentage of platelets per field of view displaying actin nodules. Data are means±s.d. (**f**) Quantification of the surface area of spread platelets from control and WAS patients. Data are means±s.d. *P=0.02 from ANOVA and Tukey's multiple comparisons. More than 125 platelets were analysed for each control and patient sample. Scale bars, 2 μm.

Podosome formation in megakaryocytes requires the action of the protein WASp^9^,^12^. In view of the similarities to podosomes, we investigated whether nodule formation also requires WASp. Platelets from mouse models either lacking WASp (*WASp* KO)^28^, or expressing a mutant form of WASP that has a critical tyrosine-residue mutated rendering it unable to be activated (*WASp* Y293F) 41, were spread on fibrinogen and stained with phalloidin. Control mouse platelets displayed nodules as previously described (Fig. 7a,b and Supplementary Fig. 6). However, nodules were absent in *WASp* KO and *WASp* Y293F platelets, even though adhesion on fibrinogen was not altered and both filopodia and lamellipodia formation was similar to controls (Fig. 7a and Supplementary Fig. 6). Small bright actin foci were occasionally seen, but these were not observed in all cells and had a morphology that was distinct from nodules (Fig. 7a). Measurement of the platelet surface area showed a small (∼10%) but significant reduction in surface area when compared with litter-matched controls (Fig. 7c).

To investigate the role of WASp in actin nodule formation in human platelets, we analysed platelet spreading from two WAS patients. Patient #1 had a c.134 C>T mutation in WASp resulting in a p.T45M missense alteration, which disrupts the interaction with WASp interacting protein (WIP)^42^. Patient #2 had a c.IVS6+2T>C mutation, which affects splicing and is predicted to give rise to WASp null platelets^43^,^44^. Both sets of patient platelets were able to adhere and spread on fibrinogen but actin nodules were absent (Fig. 7d,e). Furthermore, quantification of spreading showed a significant reduction in platelet surface area for patient #2 (WASp null; 21.5±10.5 μm^2^ versus 25.0±13.2 μm^2^ for control, *P*=0.02. Data are mean±s.d. analysed by ANOVA and Tukey's multiple comparison) and a reduction, albeit non-significant, for patient #1 (22.2±11.7 μm^2^ versus 25.0±13.2 μm^2^ for control, *P*=0.09) (Fig. 7f). Taken together these data indicate that WASp is required for the formation of nodules in both mouse and human platelets and also contributes to spreading.

### WASp recruits ARP2/3 complex to form actin nodules

**Figure 8:**
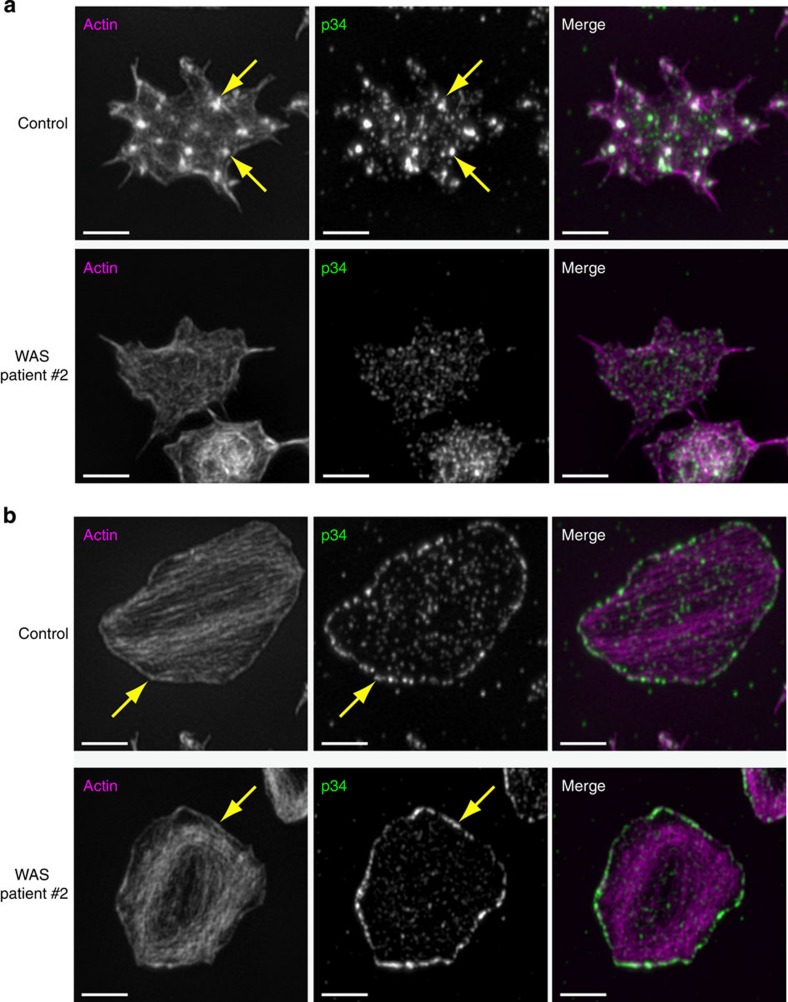
Comparison of Arp2/3 localization in control and WAS patient platelets. (**a**) SIM images of human platelets stained with Alexa488-phalloidin (left panel) and Alexa568-anti-p34 (middle panel) and the merged image (right panel, actin=magenta, p34=green). The localization of ARP2/3 complex at actin nodules can be observed in control platelets (top row, arrows indicate two examples); however, in WASp platelets no actin nodules and therefore no bright foci of ARP2/3 complex were observed. (**b**) The localization of ARP2/3 complex at lamellipodia in fully spread platelets is the same in both control and WAS patient samples indicating that ARP2/3 complex activity downstream of other NPFs is unaffected by the loss of WASp. Scale bars, 2 μm.

In light of the key role for WASp in nodule formation, we characterized the role of the ARP2/3 complex in actin nodule formation. Pre-treatment of mouse platelets with an inhibitor of ARP2/3 activity completely blocked spreading and actin nodule formation on a fibrinogen-coated surface (Supplementary Fig. 7 and Supplementary Movie 3). Although this indicated that ARP2/3 is required for actin nodule formation, this blockage could result from inhibition of spreading *per-se*. To understand the interplay between WASp and ARP2/3 in terms of nodule formation, control and WAS patient platelets were labelled for p34, a subunit of the ARP2/3 complex, to determine whether localization was affected in cells which could not make nodules. In control platelets displaying nodules (Fig. 8a, top row), ARP2/3 was seen as small punctate foci throughout the platelet, with larger and more intense foci observed at nodules (Fig. 8a, arrows). However, in WAS patient samples, which did not form nodules, no bright foci of ARP2/3 labelling were observed, whereas the small punctate foci across the platelet appeared unaltered (Fig. 8a, bottom panel). In fully spread human platelets lacking nodules, the pattern of ARP2/3 was the same in both control and WAS patient samples, that is, the small punctate foci of p34 were observed across the platelet with an enrichment of ARP2/3 and actin at the lamellipodia (Fig. 8b, arrows). Taken together these data are consistent with a model in which nodule formation is dependent on the activity of WASp, which recruits and activates ARP2/3 complex. However, ARP2/3 recruitment and activity at other parts of the cell (for example, lamellipodia) is unaffected by the absence of WASp or the presence of functionally inactive WASp.

### WASp is required for platelet aggregate formation under flow

**Figure 9:**
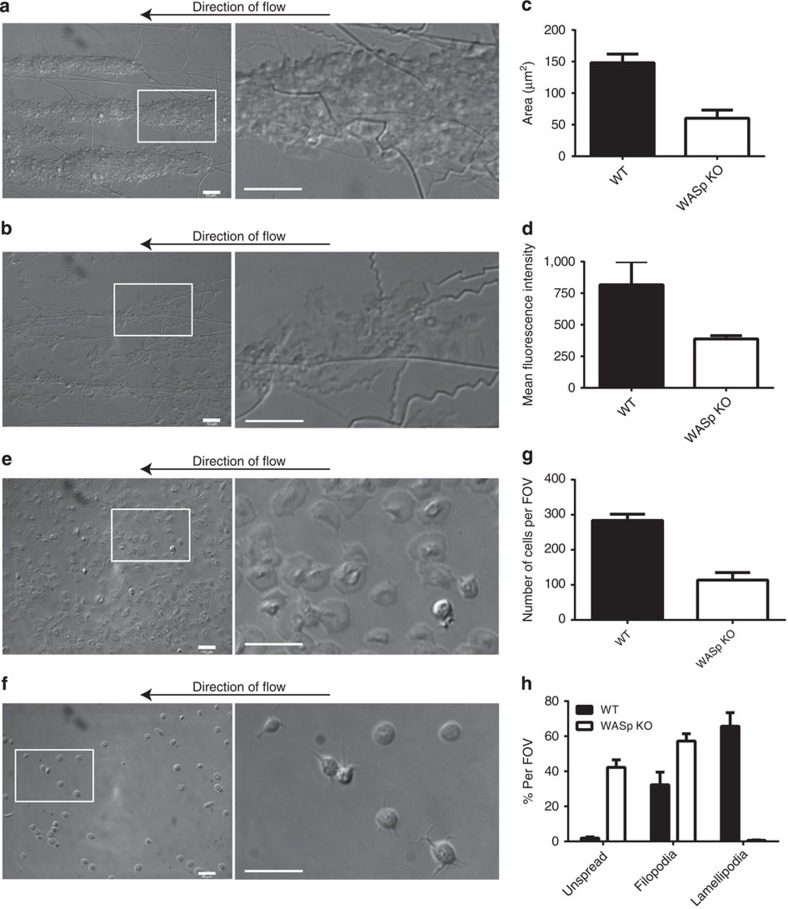
Effect of WASp KO on platelet adhesion and aggregation under flow conditions. Differential interference contrast (DIC) microscopy images of a representative field of view (FOV) from (**a**) WT and (**b**) WASp-KO mouse blood flowed over collagen-coated capillaries at a shear rate of 3,000 s^−1^ (left panel) with an enlargement of the boxed region (right panel). The failure of WASp-KO platelets to form aggregates can clearly be seen. Quantification of the aggregates in terms of their (**c**) size and (**d**) volume. DIC images of (**e**) WT and (**f**) WASp-KO mouse blood flowed over fibrinogen coated capillaries at a shear rate of 600 s^−1^ (left panel) with an enlargement of the boxed region (right panel). The reduction in both the number of platelets adhered and the extent of their spreading is clearly observed and is quantified in (**g**) and (**h**). Scale bars, 10 μm. Bars in (**c**,**d**,**g** and **h**) represent mean±s.e.m. of 10 FOV per experiment from three independent experiments.

We hypothesize that actin nodules are adhesive structures due to their association with integrins and adhesion-related proteins. However, although platelets from WASp KO mice and WAS patients do not have nodules, they display only a mild spreading defect in static adhesion and spreading assays. To further investigate this role in adhesion, we examined whether the absence of WASp impairs platelet adhesion or aggregation under flow conditions. Mouse models of WAS exhibit a reduction in platelet count and a normal platelet size compared with the severe microthrombocytopenia associated with the human disease^28^,^41^. The latter prevents interpretation of flow studies on human platelets and so studies were undertaken on WASp-KO platelets. Whole blood from WT and WASp-KO mice was flowed over collagen at a shear rate of 3,000 s^−1^. While WT platelets adhered and formed the characteristic 3D platelet aggregates (Fig. 9a), WASp-KO platelets were only able to adhere to the collagen surface as monolayers and small aggregates (Fig. 9b and Supplementary Movie 4). Quantification of the surface area covered by the aggregates demonstrates that WASp-KO platelet aggregates covered just under 50% of the surface area of WT controls (Student's *t*-Test, *P*=0.0097, 10 fields of view from *n*=3 experiments; Fig. 9c). Using fluorescence intensity as a measure of aggregate volume, we confirmed that WASp-KO platelets formed a monolayer and significantly smaller aggregates than WT controls (*P*=0.0411; Fig. 9d). This effect is specific to a functional defect with the WASp-KO platelets and not due to the reduced platelet count as WT blood with platelet count matched to those of the WASp KO platelets displayed normal aggregate formation at 3,000 s^−1^ (Supplementary Fig.8). As platelet–platelet interactions appeared to be affected by the absence of WASp, we performed low shear flow (600 s^−1^) over fibrinogen, which mediates platelet–platelet adhesion (Fig. 9e,f and Supplementary Movie 5). We observed a greater than 50% reduction in adhesion in the WASP KO compared with the WT and those platelets that did adhere were less spread (Fig. 9g,h). Taken together these results support the idea that nodules play a role in both platelet adhesion to the ECM substrate and fibrinogen-mediated platelet-platelet interactions. Thus, this indicates that adhesion via actin nodules is important for platelet capture under high shear flow conditions.

## Discussion

This study has used cutting-edge microscopy to characterize actin nodule structure and function, in both human and mouse platelets, at high spatial and temporal resolution. dSTORM and SIM provide a detailed image of protein localization in platelets to within ∼20 and ∼100 nm, respectively, which is a considerable advance over conventional light microscopy. In addition, dSTORM has enabled quantitative analysis of protein distribution. This has revealed that nodules in human platelets are composed of up to four actin centres linked by actin bundles and display differential organization of adhesion-related proteins. Furthermore, using platelets from WAS patients and a mouse model of the disease, we demonstrate that nodules are WASp-dependent structures and provide evidence that they are important for platelet aggregate formation and stability under flow conditions. We propose that the loss of nodules in platelets contributes to pathological bleeding in WAS patients.

The enhanced resolution provided by SIM and dSTORM, and studies on mutant mice, has allowed us to conclude that nodules are platelet podosome-like structures formed during the early stage of spreading. We were able to rule out that nodules represent an alternative, early adhesion-related structure, namely SICs^18^, through the demonstration that they do not co-localize with the SIC markers RACK1 or RNA and the distribution of actin is different^18^. Nodules, like podosomes, have an actin-rich core and a distinct ring of proteins^16^. In addition, nodule formation is dependent on WASp and ARP2/3 activity. However, nodules are distinct from podosomes on the basis of their relatively small size and fast turnover rate (∼10–40 s compared with 5–15 min for megakaryocyte podosomes^12^). A further property of podosomes is their ability to interact with and degrade the ECM. Megakaryocyte podosomes have been shown to use MMPs to degrade the underlying substrate and this, coupled with actin polymerization at the podosome core, provides the force necessary to protrude through the basement membrane to extend proplatelets into the bloodstream^12^. Interestingly WASp-KO megakaryocytes, which do not form podosomes, are unable to degrade the substrate and this may explain the release of platelets in the bone marrow^9^,^12^. In contrast, MMP inhibition does not affect nodule formation and nodules are not involved in substrate degradation. This difference is consistent with the shorter lifetime of nodules versus megakaryocyte podosomes.

Super-resolution microscopy has been applied to the study of podosomes to provide detail on their nanostructure^45^,^46^. dSTORM imaging of podosomes in dendritic cells (DCs) found that podosomes were interconnected by actin bundles radiating from the dense actin core^46^, bearing a strong resemblance to the actin-based interconnections of nodules in platelets. In DCs, the integrin αMβ2 was homogenously distributed as ‘islets' over the surface of the cell except at podosomes where it was completely excluded from the actin core^46^. In osteoclasts, integrins have been reported to localize to different regions of the podosome, with β3 integrins tending to localize to the outer ring while β1 localizes to the core (reviewed in ref. 16). Therefore, nodules are not alone in showing a distinct pattern of integrin organization relative to their inner core. In podosomes, this exclusion has been linked to regions of the membrane that are in close contact with the ECM^46^ and presumably is due to physical exclusion of surface molecules. In this study we observe an increase in the apparent size of actin nodules during their lifetime and have shown that this increase is due to both increased actin polymerization (this study and ref. 24) and movement of the nodule closer to the substrate. This actin polymerization-induced downward movement of the membrane towards the substrate could explain the physical exclusion of integrins from these regions^47^.

**Figure 10:**
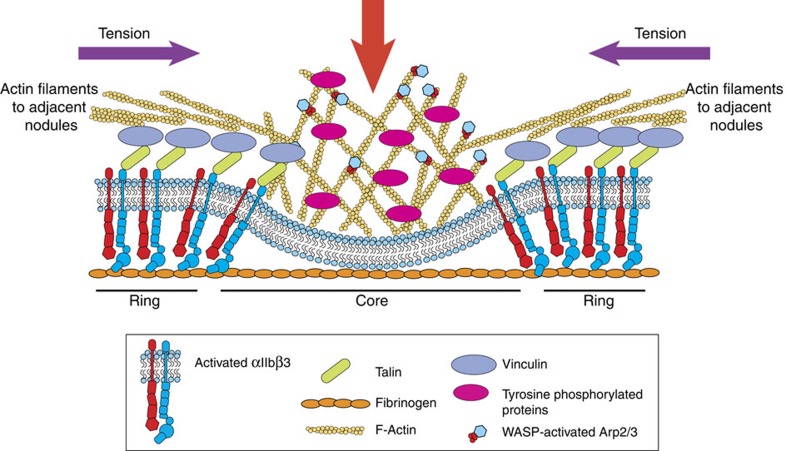
Schematic showing structural organization of the actin nodule. Actin nodules are an interconnected array of structures that consist of (i) a central WASp—ARP2/3 dependent actin core, which is rich in tyrosine phosphorylated proteins, (ii) a region under the core which is depleted of αIIbβ3 integrin, (iii) a ring structure containing talin and vinculin and (iv) actin filaments which radiate out to other nodules. We hypothesize that the actin polymerization-driven downward movement of the membrane (red arrow) causes the physical exclusion of integrin from the centre of the nodule. This generates tension in the actin filaments linking adjacent actin nodules (purple arrows) and so plays a mechano-sensory role important for maintaining platelet adhesion in blood flow.

The adhesion-related proteins talin and vinculin are characteristic of the ring structure of podosomes in many cell types including megakaryocytes^16^. However, in DCs, talin and vinculin have been shown to have a differential patterning around the podosomes. Talin exhibited a similar distribution to the integrin (homogenously distributed but depleted from the podosome core), whereas vinculin was enriched around the actin core and along the radiating actin bundles^46^. In platelets, using SIM, we see both talin and vinculin throughout the platelet with enrichment of both around the nodule core. Both talin and vinculin have been shown to be important for mechano-sensing^48^,^49^. This role is achieved through the ability of talin to link the actin cytoskeleton to activated integrins, which supports adhesion to the ECM^50^ and platelet–platelet interactions^51^. The link between actin and integrins is reinforced by the binding of vinculin. The vinculin head domain binds to sites along the talin rod domain, and its tail domain binds to actin^52^,^53^. The role of vinculin in reinforcing, rather than being essential for mechano-sensing, is highlighted by the increase in bleeding times observed in vinculin-null mice despite platelet function being unaffected^54^. The dynamic, interconnected nature of actin nodules and the organization of integrins and adhesion-related proteins at these structures lead us to predict that actin nodules play a role in mechano-coupling between platelets and the substrate or platelets and other platelets as proposed in Fig. 10. Furthermore, the observation that moesin and α-actinin molecules that are known to link actin filaments to membranes and to be involved in mechano-sensing, respectively^55^,^56^,^57^, are enriched at actin nodules (Supplementary Fig. 9), provides further (indirect) evidence for this hypothesis.

Our lab has previously shown that SFK and actin polymerization is required for stable platelet aggregate formation under flow conditions^58^. We have shown here that the actin polymerization required for nodule formation is dependent on the WASp-ARP2/3 axis as WASp-KO platelets do not have nodules. However, ARP2/3 activity downstream of other NPFs (for example, Scar/WAVE^59^) is unaffected as WASp-null platelets are still able to form lamellipodia and correctly localize ARP2/3 to lamellipodia in spread platelets. Our data show that while WASp-KO platelets have a small defect in spreading under static assays, it is under conditions mimicking arterial shear rates that the major effect is observed. WASp-KO platelets do not adhere as efficiently to collagen or fibrinogen under flow conditions and form only monolayers and small aggregates on collagen at high shear rates. Consistent with this, nodules can be seen in platelets spreading on other substrates such as collagen or VWF (plus botrocetin)^24^. The formation of nodules, however, is most prevalent in platelets spreading on fibrinogen. Fibrinogen is a ligand for the αIIbβ3 integrin and is primarily involved in platelet–platelet interactions in thrombus formation. This dependency on WASp implicates a role for actin nodules in platelet-substrate and platelet-platelet adhesion under shear and may underlie the increase in bleeding in WAS patients and unstable primary platelet plugs in mouse tail bleeding assays in the absence of WASp^31^.

In summary, we have provided strong evidence for the role of WASp in platelets. We show that it is required for the formation of actin nodules, structures, which share some similarities with podosomes, and demonstrate that it is required for robust aggregate formation under flow conditions.

## Methods

### Reagents

Human Fibrinogen (Plasminogen, von Willebrand Factor and Fibronectin depleted) was obtained from Enzyme Research Laboratories (Swansea, UK). Horm collagen was obtained from Takeda (High Wycombe, UK). Alexa488-fibrinogen, Alexa488-phalloidin, Alexa568-phalloidin, Anti-rabbit-Alexa568 (A-11036), Anti-mouse-Alexa568 (A-11031) and Anti-mouse-Alexa647 (A-21235) secondary antibodies were purchased from Life Technologies (Paisley, UK). The 35-mm uncoated glass bottomed dishes with No1.5 coverslips were obtained from MatTek Corporation (Massachusetts, USA). Monoclonal Anti-vinculin (clone hVIN-1, V9131), Anti-Talin (Clone 8d4, T3287), 10% neutral buffered Formalin solution, monoclonal Anti-Moesin (Clone 38/87, M7060) and anti-α-actinin (A2543) were purchased from Sigma (Poole, UK). Monoclonal Anti-Phosphotyrosine (clone 4G10, 05-1050), Anti p34-Arc(ARPC2, 07-227) and ARP2/3 complex inhibitor 1 (CK666) were purchased from Merck-Millipore (Middlesex, UK). Monoclonal Mouse Anti-Human CD41 (αIIb)-FITC (clone 5B12, F708801-2) was obtained from Dako UK (Cambridgeshire, UK). Mouse Anti-RACK1 (clone20/RACK1) was purchased from BD Transduction Laboratories (Oxford, UK, 610178). Sytox-Green was purchased from Life Technologies (Paisley, UK). GM6001 was purchased from Merck Millipore (Watford, UK). Unless specified otherwise, antibodies were used at a dilution of 1:250 of the supplied stock solution. All fluorescent secondary antibodies and phalloidin were used at a 1:300 dilution.

### Mice

The 9- to 12-week-old C57Blk6, Lifeact-GFP^60^, WASp KO^28^ or WASp Y293F^41^ mice were maintained in IVCs under 12 h light/dark cycle at a constant temperature of 20 °C with food and water given *ad libitum* at BMSU, Birmingham University, UK. All experiments were performed in accordance with UK laws (Animal [Scientific Procedures] Act 1986) with approval of local ethics committee (Birmingham Animal Welfare and Ethical Review Board—AWERB) under a Home Office approved project licence.

### Platelet preparation

Human blood samples for platelet isolation were donated by healthy volunteers under the following licence: ERN_11-0175 ‘The regulation of activation of platelets' (UoB). Washed platelets were prepared as previously described^4^. In brief, blood was drawn by venipuncture from healthy volunteers into sodium citrate and acid/citrate/dextrose. Platelet-rich plasma (PRP) was prepared by centrifugation of whole blood at 200 × *g* for 20 min. The platelets were then isolated from PRP by centrifugation at 1,000 × *g* for 10 min in the presence of prostacyclin (0.1 μg ml^−1^). The pellet was resuspended in modified Tyrodes buffer (129 mM NaCl, 0.34 mM Na_2_HPO_4_, 2.9 mM KCl, 12 mM NaHCO_3_, 20 mM HEPES, 5 mM glucose, 1 mM MgCl_2_; pH 7.3) containing 0.1 μg ml^−1^ prostacyclin. The platelets were washed once via centrifugation (1,000 × *g* for 10 min) and resuspended at the desired concentration with modified Tyrodes buffer.

Washed platelet samples of mouse were prepared as previously described^4^. In brief, blood was drawn from CO_2_ terminally anaesthetized mice from the vena cava and taken into 100 μl of acid/citrate/dextrose. PRP was obtained by centrifugation at 200 × *g* for 6 min. Washed platelets were prepared via centrifugation of PRP at 1,000 × *g* in the presence of prostacyclin (0.1 μg ml^−1^) for 6 min. The pellet was resuspended in modified Tyrode buffer to the desired platelet level.

### Wiskott–Aldrich syndrome patients

This study was approved by the National Research Ethics Service Committee West Midlands—Edgbaston (REC reference: 06/MRE07/36) and participants and controls gave written informed consent in accordance with the Declaration of Helsinki.

**Patient #1** c. 134C>T *WASP* mutation predicting a p. T45M missense alteration. This disrupts binding of WASp to WIP binding leading to increased WASp degradation^42^. This patient expressed very low levels of WASp.

**Patient #2** c. IVS6+2T>C mutation that affects splicing and is predicted to give rise to WASp null platelets^43^,^44^. This patient expressed no WASp.

### Platelet spreading and staining

For fixed cell imaging experiments, washed and rested platelets were diluted to 2 × 10^7^ cells ml^−1^ in modified Tyrodes buffer and allowed to spread on glass bottomed dishes coated with fibrinogen (100 μg ml^−1^) for 30 min at 37 °C. Adhered cells were rinsed briefly twice with phosphate buffered saline (PBS) and fixed for 10 min with 10% formalin solution. Fixed samples were washed three times with PBS and also between each step of the staining procedure. Fixed platelets were treated with 50 mM NH_4_Cl for 10 min to quench residual formalin fluorescence, permeabilized with 0.1% Triton X-100 in PBS for 5 min and then stained with antibodies or phalloidin as required. Samples for N-SIM imaging were mounted in Hydromount and stored at room temperature. Samples for dSTORM imaging were stored in PBS at room temperature and protected from light until imaged. When required, platelets were treated with the ARP2/3 inhibitor, CK666 (10 μm)^61^,^62^, or Monoclonal Mouse Anti-Human CD41 (αIIb)-FITC for 10 min at 37 °C before spreading. Following imaging (see below), platelet surface area was measured using ImageJ (NIH, Bethesda, USA). Data were analysed using Graphpad Prism v6 (Graphpad, California, USA).

### Treatment of platelets with the MMP inhibitor GM6001

Washed human platelets at 2 × 10^7^ cells ml^−1^ were treated with 100 μM GM6001, or the same volume of DMSO as a vehicle control, for 10 min at 37 °C before being spread for 30 min on Alexa488-fibrinogen-coated coverslips. Platelets were then fixed in formalin, permeabilized, stained for actin with Alexa568-phalloidin and imaged by z-stack confocal microscopy using a Leica SP2 inverted confocal with the 63 × objective. Five fields of view (59 × 59 μm) were taken per treatment, per experiment (*n*=3; a total of >360 platelets per treatment). Platelet number per field of view, surface area and number of nodules μm^−2^ were calculated using the Nikon NIS Elements v4.1 software.

### Flow-adhesion assay

Blood from three WT and three WASP-KO mice was drawn into sodium heparin (10 U ml^−1^) and PPACK (D-phenylalanyl-L-prolyl-L-arginine chloromethyl ketone–40 μM). Glass capillaries, 0.1 × 1 mm internal diameter (Camlab, Cambridge, UK) were coated with 100 μg ml^−1^ Horm collagen or 100 μg ml^−1^ fibrinogen overnight at 4 °C then blocked with 5 mg ml^−1^ heat-inactivated BSA in PBS for 1 h at room temperature. The capillaries were mounted on the stage of an inverted microscope (DM IRB; Leica), connected to a Harvard pump driven flow system and rinsed through with PBS to remove the BSA block. The blood was pre-incubated with DiOC6 (3,3′-dihexyloxacarbocyanine iodide–2 μM) for 10 min before perfusion through the chamber at 37 °C at the shear rate and duration indicated in the text and figure legend. Platelet aggregates were washed with modified-Tyrode's buffer at the same flow rate as the blood for 5 min and then immediately imaged by DIC and z-stack fluorescence microscopy on a Zeiss Axiovert 200 M with a 63 × objective. Ten fields of view, along the length of the capillary, per flow experiment were imaged. For flow over Horm collagen platelet aggregate formation was assessed in ImageJ by thresholding the fluorescence image to identify the aggregates. The mean surface area (μm^2^) of individual thrombi was assessed in both WT and KO. The mean fluorescence intensity of the aggregates was then calculated as a measure of the aggregate volume. To test the effect of reduced platelet count on platelet aggregate formation, samples of WT mouse blood had the platelet count recorded before being split in half; one aliquot was centrifuged at 200 × g. for 6 min to produce (PRP) and a red blood cell fraction. The PRP had PGI_2_ (0.1 μg ml^−1^ PGI_2_ per ml PRP) added and was spun for a further 6 min at 1,000 × g to pellet the platelets. This platelet-poor plasma (PPP) was added back to the red cell fraction to generate reconstituted blood with a reduced platelet count. The second aliquot was left untreated, except for the addition of an equivalent concentration of PGI_2_. WT and platelet-depleted blood were then mixed in the appropriate ratios to give blood with platelet counts ranging from 1,200 to 282 × 10^3^ platelets μl^−1^. For analysis of the fibrinogen flow data the number of platelets per field of view (10 per experiment) were manually counted using the ImageJ plugin ‘Cell counter'. Individual platelets were then scored as being (1) unspread, (2) having filopodia, (3) having lamellipodia and expressed as a percentage of the total platelets counted in that field of view. Student's *t*-tests were carried out in Graphpad Prism v6 to assess significance.

### Total internal reflection fluorescence microscopy

Live Lifeact-GFP platelets were imaged using an Olympus IX81 Inverted microscope with a 60x Plan Apo 1.49NA oil-immersion objective and a Hammamatsu ORCA-R2 C10600 12-bit CCD. Samples were excited with a 491-50 Diode type laser. For the dual TIRF—Epifluorescence live cell imaging of actin nodules a 150 × objective (UAPON 150XOTIRF, 1.45 NA, Olympus) was used with the same Olympus TIRF microscope. The 491-nm laser was set at the same power and gain, with the angle of the laser altered to achieve TIRF (with a penetration depth of 100 nm) and then immediately switched to widefield (Epi) illumination. Images were taken every 2 s and drift in Z was minimized with Olympus Z-drift correction (ZDC). Images were acquired using Xcellence Advanced Live Cell Imaging System 1.1. Post-imaging analysis was performed using Nikon NIS Elements v4.1 and ImageJ 1.47v.

To calculate the nodule movement in Z, regions of interest (ROIs) were drawn around nodules that were seen to appear and disappear within the timeframe of the movie. The intensity of both the TIRF and Epi channels were measured and plotted against time. The vast majority of nodules showed a much greater increase in intensity in TIRF than in Epi, indicating movement towards the coverslip. With the assumption that the contribution to intensity from actin polymerization was minimal the movement in Z can be calculated from the TIRF intensity values over time using equations 1, 2, 3, 4,^39^:


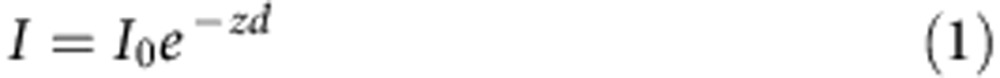


Where *I*_0_ is the intensity of the laser, *z* the distance from the coverslip and *d* the penetration depth (a function of TIRF angle and refractive index). Assuming that pixel value is proportional to the intensity of the TIRF field then


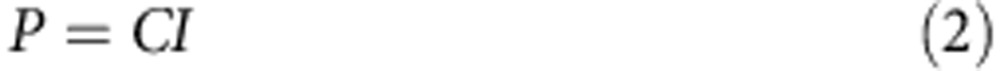


Where *P* is the pixel intensity value and *C* is a constant, which depends on detector sensitivity, detector gain and fluorophore concentration. We know that these factors remain constant between TIRF and Epi images then *C* remains the same for different time points. Therefore, the ratio of pixel intensities at two time points equals


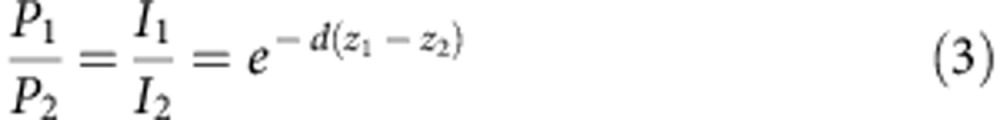


This rearranges to


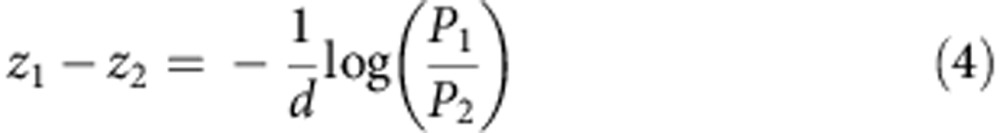


Using this equation the movement in Z of an actin nodule between each time point was calculated. From this the overall movement of an actin nodule, from first appearance to disappearance, was plotted against time. Values for each nodule were calculated in Excel 2010 and graphs plotted in Graphpad Prism v6.

### Structured illumination microscopy

Samples were imaged on a Nikon N-SIM system consisting of a Ti-E stand with Perfect Focus, 100 × 1.49 NA TIRF objective lens, Nikon LU5 laser bed (488 and 561 nm laser lines), 3D SIM grating and Andor DU897 EMCCD camera. Images were captured using Nikon NIS Elements v4.1 and reconstructed using slice reconstruction in NIS elements. Post-imaging analysis was performed using NIS Elements v4.1 and ImageJ 1.47v.

### STORM imaging and cluster analysis

Samples were imaged on a Nikon N-STORM system in dSTORM mode consisting of a Ti-E stand with Perfect Focus, 100 × 1.49 NA TIRF objective lens, Agilent MLC400 high power laser bed (647 nm laser line) and Andor DU897 EMCCD camera. Samples were imaged in a PBS buffer containing MEA (100 mM) and GLOX (10% v/v) to induce fluorophore blinking^63^. Twenty thousand frames were captured using Nikon NIS Elements v4.1 and reconstructed using STORM analysis module 1.1. Samples were drift corrected and rendered using Gaussian rendering. Spots present in the final reconstructed images represent individually identified fluorescent blinking events. Post-imaging analysis was performed using Nikon NIS Elements v4.1. For information: the median localization precision, a measure used to assess quality of super-resolution images^64^, routinely achieved for 4G10 imaging was 12.8 nm (range 1.58 to 37.6 nm) and for CD41 it was 17.9 nm (range 2.8–66.4 nm), calculated from five images. Cluster analysis was performed on the dSTORM data in MATLAB using a custom made algorithm as described by Owen *et al.*^34^, based on Ripley's K-function and Getis and Franklin's Local Point Pattern analysis and described briefly here.

The K-function, *K*(*r*), for a molecule, *j*, within an analysed region of area A containing *n* molecules in total is defined as:


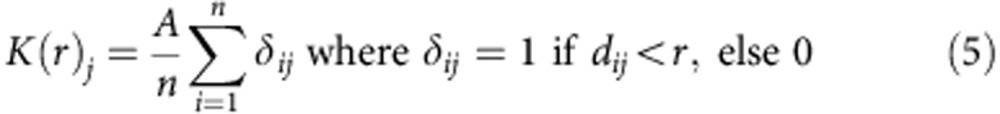


Where *d*_*ij*_ is the distance from molecule *j* to molecule *i*. In essence, this value is the number of molecules found within a radius *r* of molecule *j*, divided by the mean molecular density (n/A) of the region. Since the value of *K*(*r*) scales as *r*^2^ the K-function is typically linearized to generate the L-function such that *L*(*r*)=sqrt(*K*(*r*)/π) so:


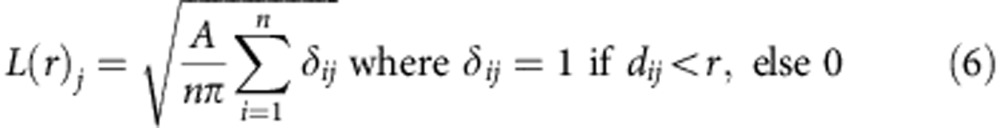


Here, the value of *L*(*r*) was computed for each molecule in a region. For analysis, the region of interest was set at 1 μm^2^, and the spatial scale analysed (*r*) was set to 50 nm. Thus,


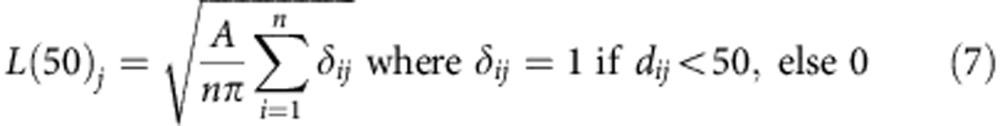


To generate a pseudo-coloured cluster map, the values of *L*(50) at each point, *j*, where interpolated onto a 5 nm resolution grid (Matlab ‘v4' interpolation algorithm). This cluster map could then have an upper and lower threshold applied to them to generate binary maps. Areas of the map below the lower threshold were defined as being holes—where the local molecular density is low relative to the mean molecular density, whereas areas above the upper threshold were defined as clusters. For integrin analysis the lower binary threshold was set at *L*(50)=20 and the upper binary threshold was set at *L*(50)=70. For pTyr analysis the upper binary threshold for clustering was set at *L*(50)=100. Twenty-five ROIs containing actin nodules and 25 without were analysed for pTyr clustering quantification. All dSTORM analyses were performed on human platelets.

### SEM prep and imaging

Platelet cytoskeletons were isolated by permeabilizing cells with PHEM buffer (60 mM Pipes, 25 mM Hepes, 10 mM EGTA, and 2 mM MgCl_2_) containing 0.75% Triton X-100 for 2 min. Cytoskeletons were then fixed with 1% glutaraldehyde in PHEM for 10 min Water washed coverslips of platelet cytoskeletons were rapidly frozen, freeze-dried, and coated with platinum and carbon. Replicas were picked up on carbon-formvar-coated copper grids and examined with a JEOL JEM-1200 EX transmission electron microscope at an accelerating voltage of 80 kV.

## Additional information

**How to cite this article:** Poulter, N. S. *et al.* Platelet actin nodules are podosome-like structures dependent on Wiskott–Aldrich syndrome protein and ARP2/3 complex. *Nat. Commun.* 6:7254 doi: 10.1038/ncomms8254 (2015).

## Supplementary Material

Supplementary Information Supplementary Figures 1-9, Supplementary Notes 1-2 and Supplementary References

Supplementary Movie 1 Three representative Lifeact-GFP mouse platelets spreading on fibrinogen surface showing the dynamic nature of actin nodules. Scale bar = 5μm. Image taken every 2 seconds.

Supplementary Movie 2 Lifeact-GFP mouse platelets spreading on fibrinogen and imaged in TIRF (left panel; green) and Epi (right panel; magenta). Laser power and detector gain setting kept constant with rapid switching between TIRF and Epi modes. Boxed region indicates the representative nodule described in Figure 6e & f. Scale bar = 5μm. Image taken every 2 seconds.

Supplementary Movie 3 Effect of 20μM CK666 on Lifeact-GFP mouse platelet spreading. The platelets in the right hand panel were treated with the inhibitor for 10 min prior to spreading on a fibrinogen surface. Scale bar = 5μm. Image taken every 2.5 seconds.

Supplementary Movie 4 Wildtype or WASp KO mouse whole blood labelled with DICO6 and flowed over Horm collagen at a shear of 3000s-1 for 1.5 minutes. Scale bar = 20μm.

Supplementary Movie 5 Wildtype or WASp KO mouse whole blood, labelled with DICO6 and flowed over fibrinogen at a shear of 600s-1 for 4 minutes. Scale bar = 10μm.
