# Supplementary material for: Platelet actin nodules are podosome-like structures dependent on Wiskott–Aldrich syndrome protein and ARP2/3 complex
Source: Nat Commun. 2015 Jun 1;6:7254. doi: 10.1038/ncomms8254 (PMC4458878; doi:10.1038/ncomms8254)
Supplement: Supplementary Information — Supplementary Figures 1-9, Supplementary Notes 1-2 and Supplementary References [file ncomms8254-s1.pdf]

Supplementary Figures

Supplementary Figure 1

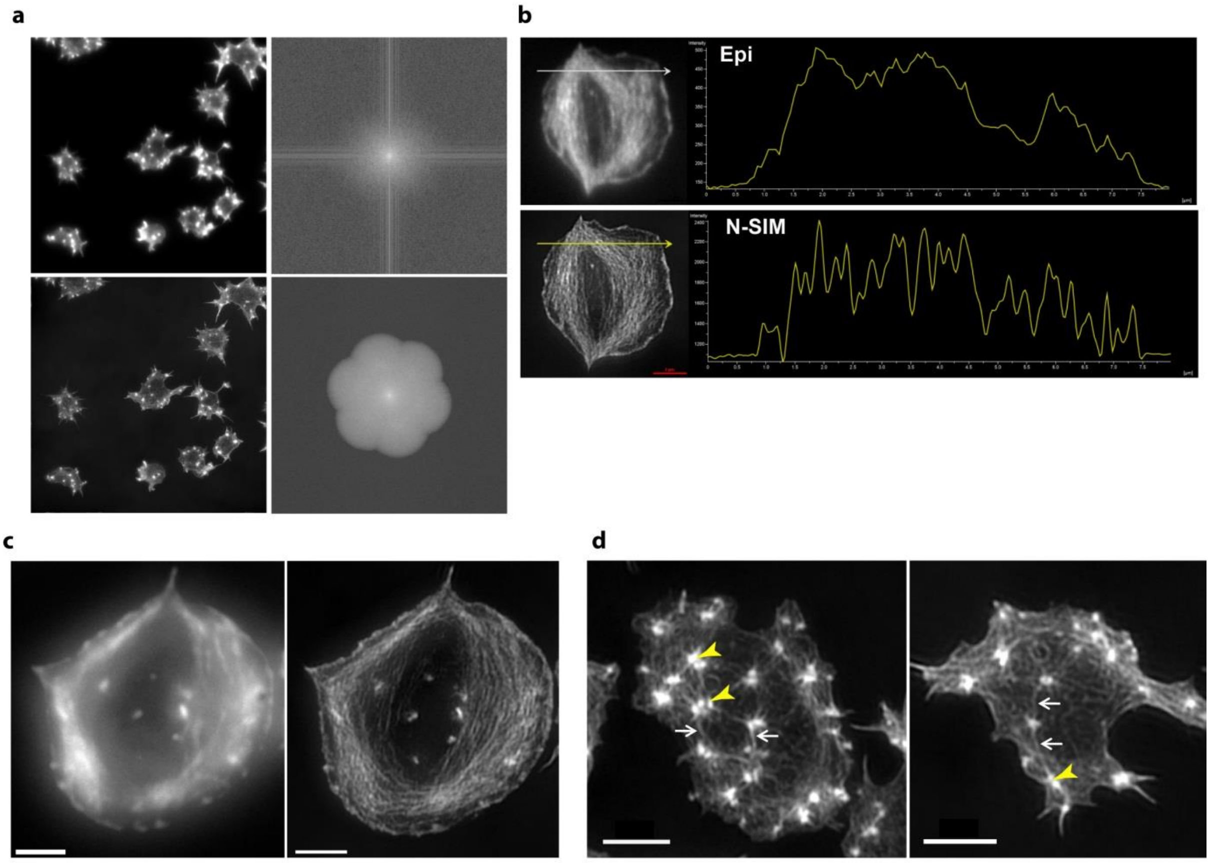

SIM imaging achieves a 2x increase in resolution over regular epifluorescence imaging.

a) Diffraction limited Epifluorescence (upper left panel) and SIM (lower left panel) images of the same human platelets stained with Alexa488-phalloidin. Fourier transforms of the SIM image data (Lower right panel) clearly shows the characteristic "petal" arrangement indicative that SIM imaging has overcome the diffraction limit as seen for the epifluorescence image (upper right panel). b) Example line intensity profiles for the same human platelet imaged by Epifluorescence and SIM imaging. Comparison of the profiles shows that SIM can clearly resolve objects that are not clear in the diffraction limited Epifluorescence image. Measurement of the full width half maxima (FWHM) for the same objects in both imaging modalities gave an average increase in resolution of 2x (0.122 ± 0.03μm for SIM compared with 0.24 ±0.03μm for epifluorescence. Data are means ± SD. c) Epifluorescence (left) and SIM (right) images of a spread human platelet demonstrating the power of SIM to resolve sub-cellular structures. F-actin bundles are clearly visible in regions of the cell, when visualised with SIM, that appear as unresolved fluorescence signal with diffraction limited Epifluorescence imaging. d) Additional representative SIM images of two further human platelets displaying actin nodules showing resolution of actin nodules into multiple foci (arrowheads) and the actin fibres interconnecting different actin nodules (arrows). Scale bar: 2 μm.

Supplementary Figure 2

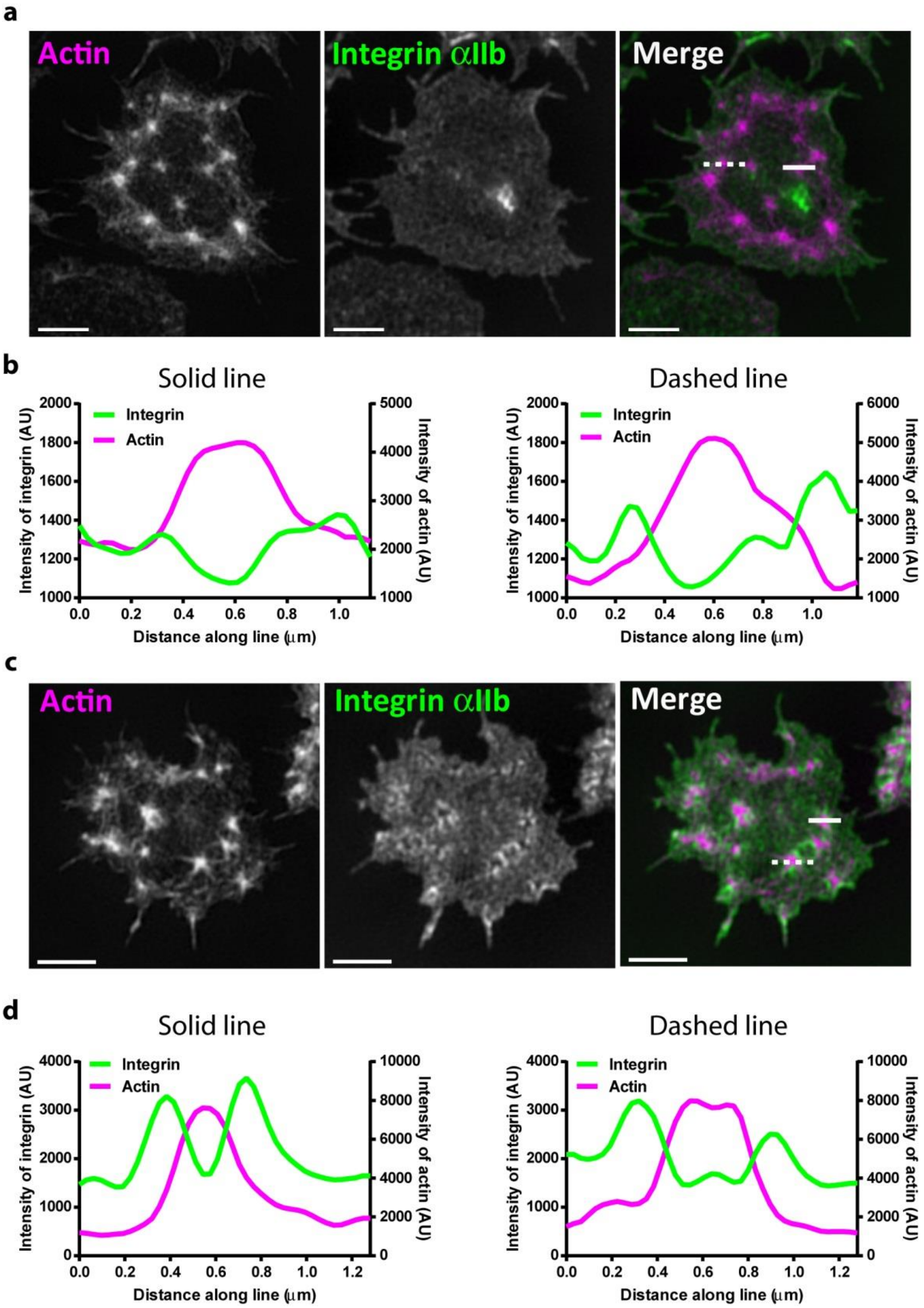

SIM imaging of integrin holes at actin nodules.

a) & c) SIM images of human platelets stained for F-actin (Left panel), αIIb integrin (Middle panel) and the merged images (Right panel, actin = magenta, integrin = green) showing further examples of the holes in the integrin labelling that is observed at actin nodules and the enrichment of integrin that is seen around some actin nodules. b) & d) Line intensity scans of actin and integrin fluorescence signal showing the integrin holes and actin enrichment observed at actin nodules. Scale bar: 2 μm.

Supplementary Figure 3

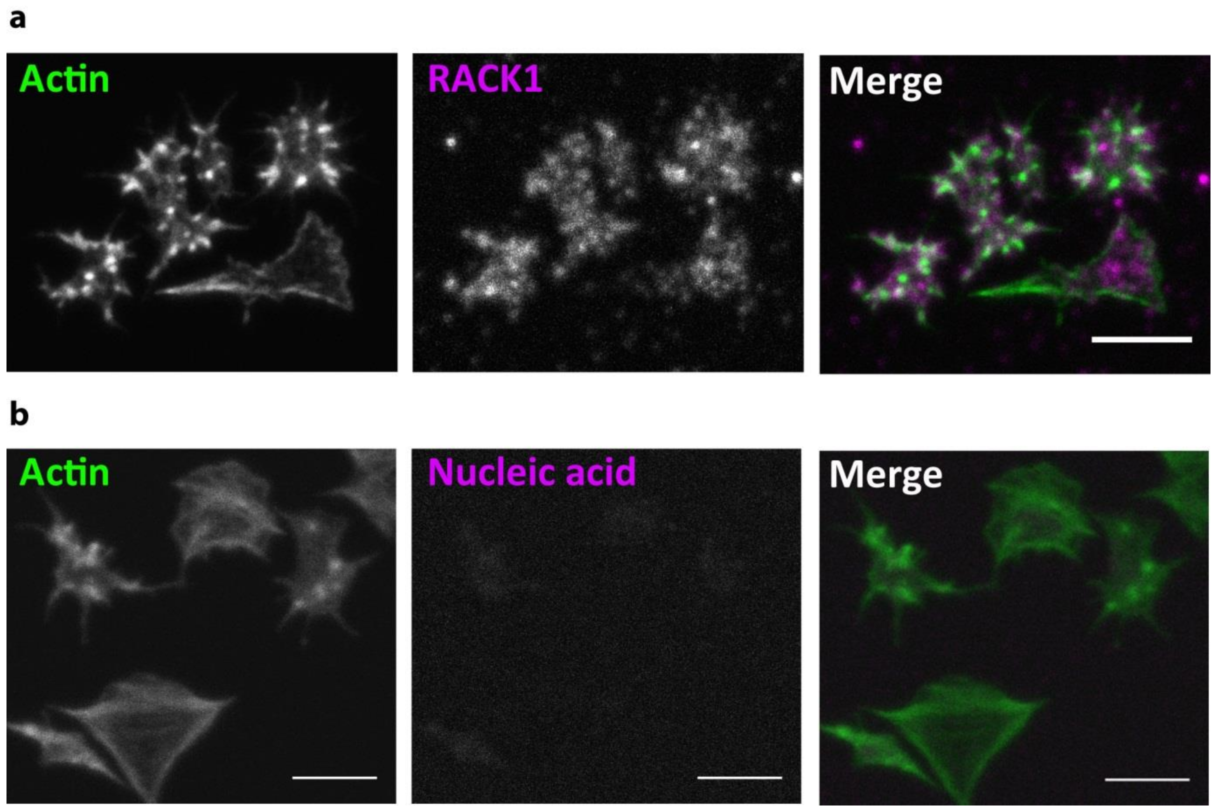

Actin nodules are not spreading initiation centres (SICs).

Confocal Z-stack images of washed human platelets spread on fibrinogen for 30 min before fixation and staining for markers of SICs. a) Actin nodules (Alexa488-phalloidin; green) do not colocalise with RACK1 (1μg ml^{-1} mouse anit-RACK1, secondary Alexa547; magenta). b) Actin nodules (Alexa568-phalloidin; green) are also not enriched in RNA (stained with a 1:1000 dilution of Sytox-green; magenta). Scale bar: 5μm. Images are representative of platelets from 3 independent experiments.

Supplementary Figure 4

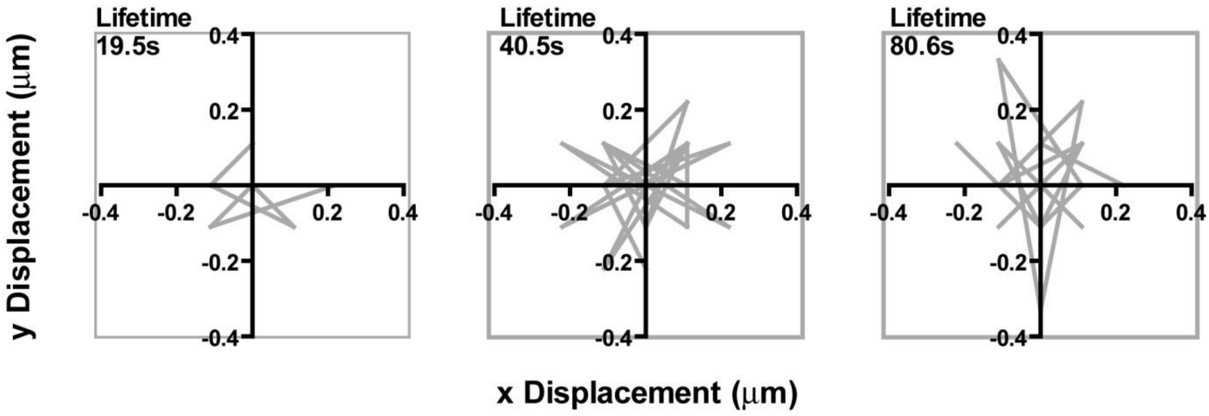

Limited mobility of actin nodules.

Representative displacement plots of three actin nodules with short (19.5 sec, left panel), medium (40.5 sec, middle panel) and long (80.6 sec, right panel) lifetimes showing that nodules do not display any directional movement and appear constrained to their site of formation.

Supplementary Figure 5

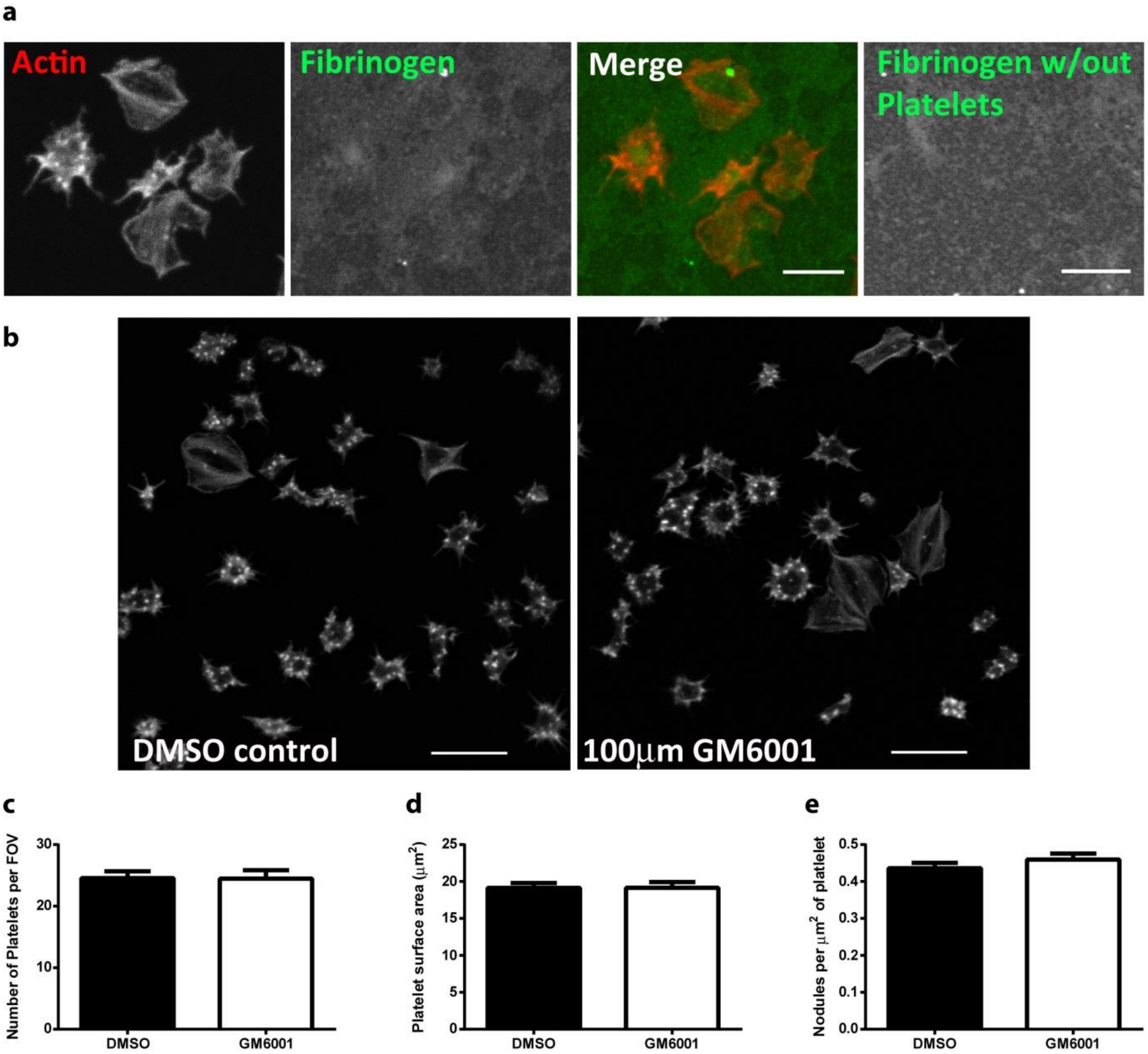

Actin nodules do not degrade fibrinogen and are not affected by MMP inhibition.

a) Confocal maximum intensity projection of human platelets spread for 30 min on fluorescently labelled fibrinogen (Alexa488-fibrinogen; green) and labelled with Alexa568-phalloidin (red). The Alexa488-fibrinogen underneath the platelets does not look any different to the fibrinogen coating where no platelets have been added. No holes are visible in the fibrinogen indicating that actin nodules do not degrade the extracellular matrix. Sale bar: 5μm. b) Confocal maximum intensity projection of washed human platelets pre-treated for 10 min with DMSO vehicle control (Left panel) or 100 μm GM6001, the broad spectrum MMP inhibitor (Right panel), before being spread for 30 min on fibrinogen the fixed and stained for actin with Alexa488-phalloidin. Sale bar: 10 μm. c) No difference was seen in the number of platelets adhering to the substrate between DMSO and GM6001 treatment. d) Platelet surface area was also unaffected as was the number of nodules per μm^2 of platelet area (e). Results are taken from n=3 independent experiments with a total of >360 platelets analysed per treatment.

Supplementary Figure 6

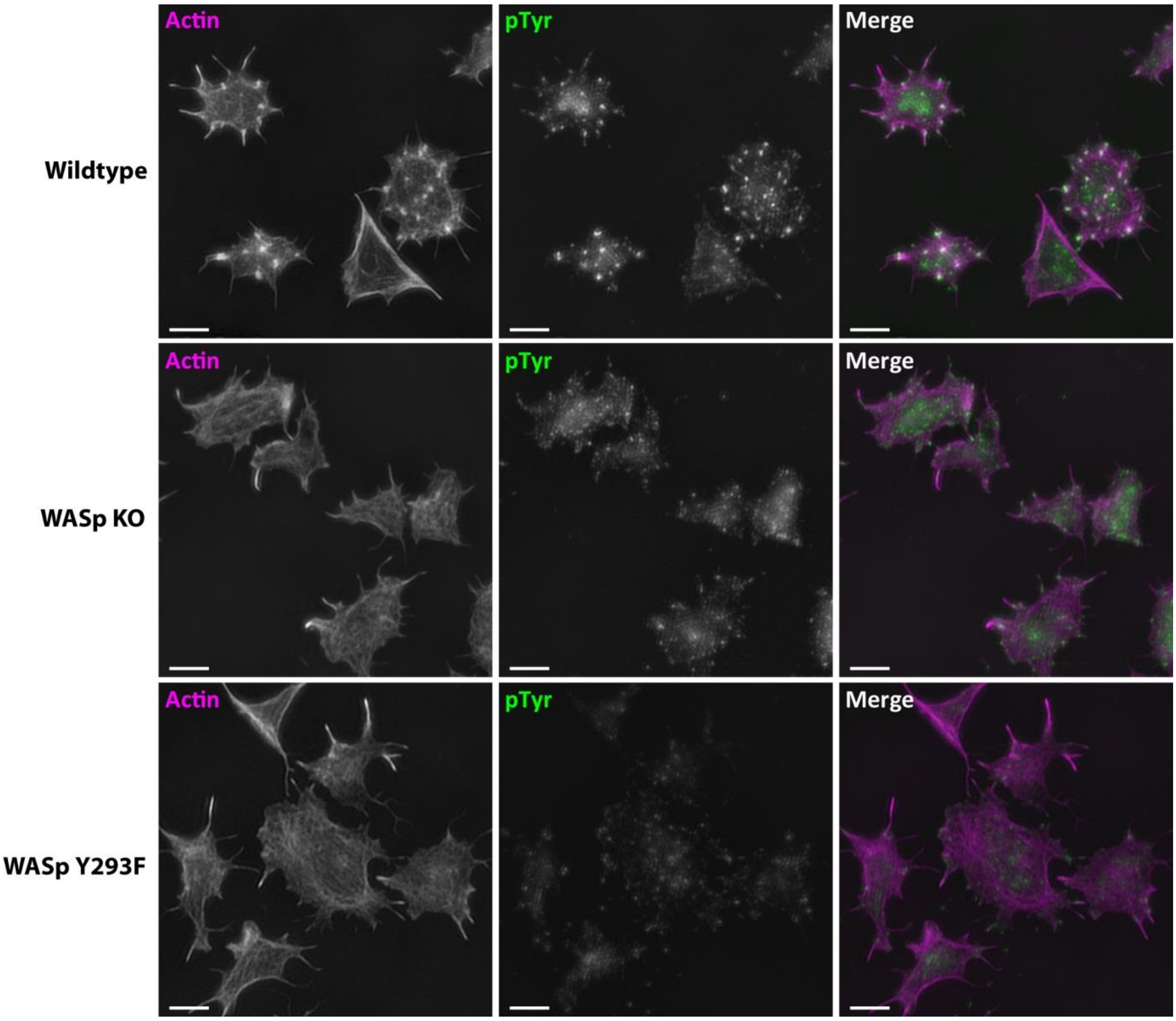

pTyr labelling in WT, WASp KO and Y293F mouse platelets.

SIM images of mouse platelets stained for F-actin (Left panel), pTyr (Middle panel) and the merged images (Right panel, actin = magenta, pTyr = green) from wildtype (Top row), WASp knockout (Middle row) and WASp Y293F (Bottom row). Images indicate that actin nodules were not formed in either the knockout or mutant WASp platelets during spreading and consequently they did not show the bright clustered foci of protein tyrosine phosphorylation as was observed for wildtype platelets. Scale bar: 2μm.

Supplementary figure 7

a

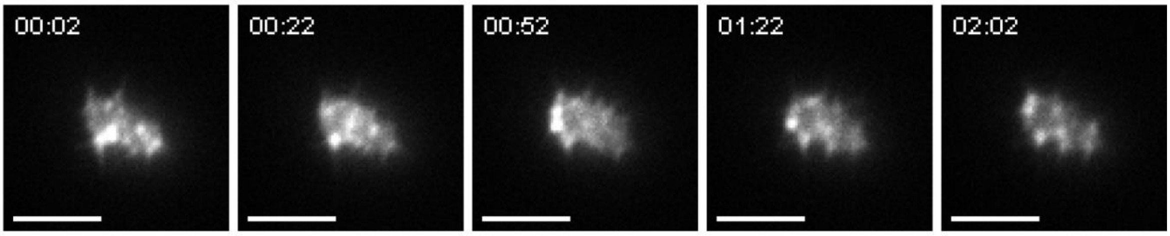

b

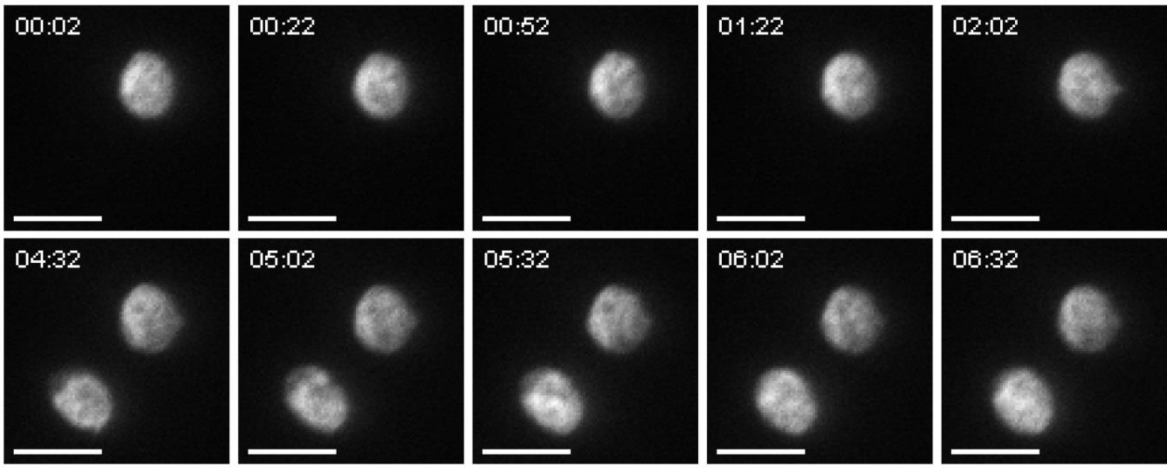

c

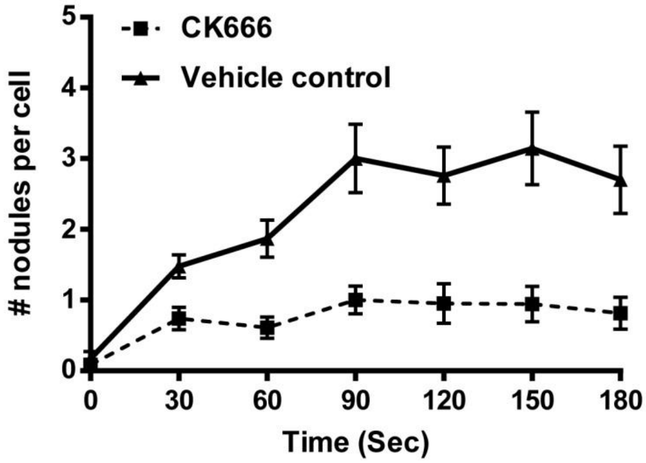

Effect of ARP2/3 inhibition on actin nodule formation.

a) Frames taken from time-lapse TIRF movies of a representative Lifeact-GFP mouse platelets spreading on fibrinogen (See supplementary movie 2) showing the dynamic nature of actin nodule formation and turnover. Time stamp (Top left) = min:sec. b) Platelets were pre-treated with 20 μm CK666 for 10 min prior to spreading which blocked spreading and actin nodule formation. Time stamp (Top left) = min:sec. c)Quantification of the mean number of actin nodules per platelet over time from control (Solid line) and CK666 treated (Dashed line) platelets. Data represent means ± standard error of the mean. Scale bar: 5 μm.

Supplementary Figure 8

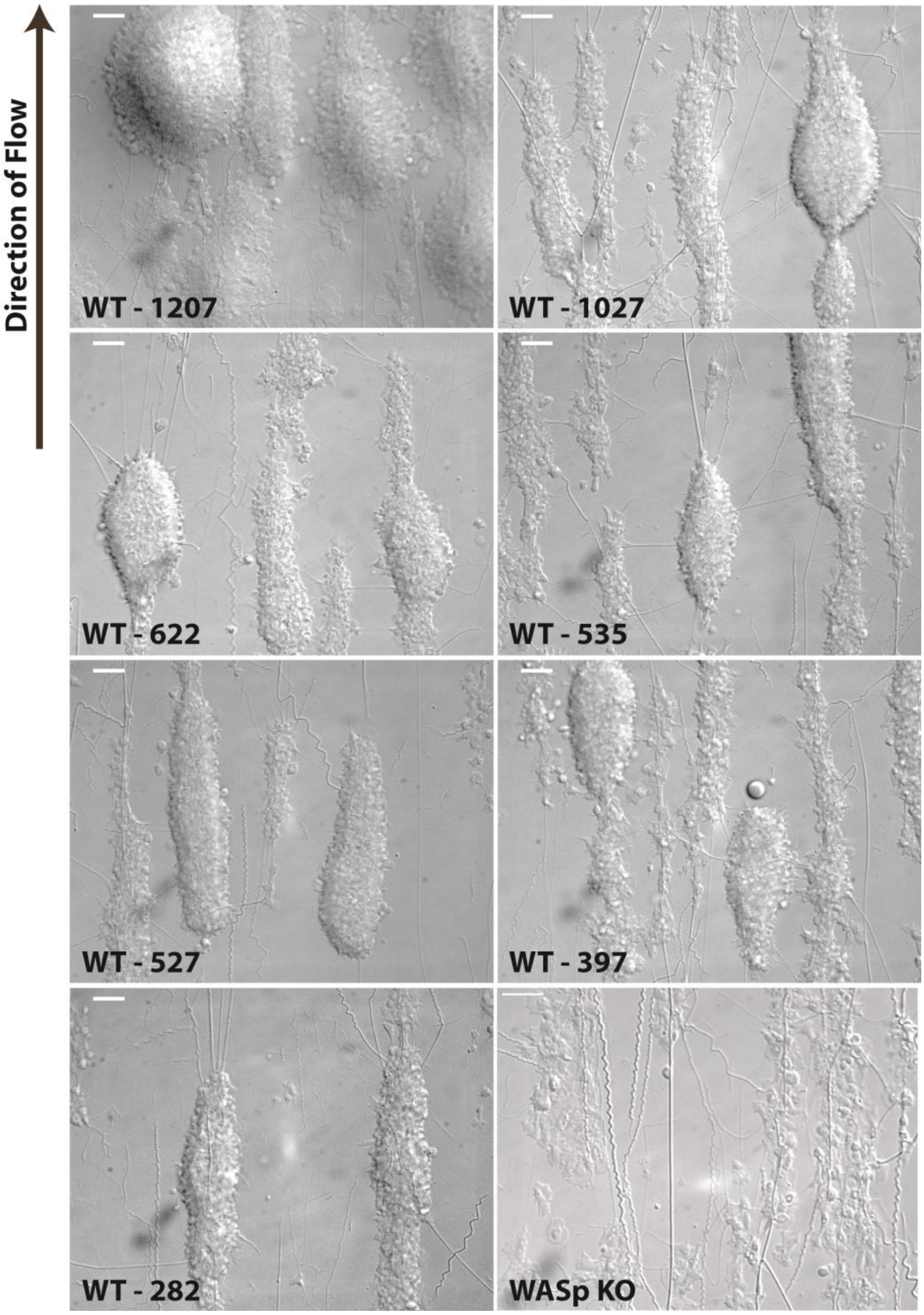

Effect of platelet count on platelet aggregation under flow.

Differential Interference Contrast (DIC) microscopy images of a representative field of view from WT mouse blood flowing over collagen coated capillaries at a shear rate of $3000s^{-1}$. Platelet counts were adjusted to give a range from ~1200 to ~300 platelets mm^3. The platelet counts for each sample are shown in the lower left corner. Even with a reduced platelet count, robust aggregates were still able to form compared to the single layer of adhered platelets observed in the WASp KO mouse samples (lower right image). This indicates that reduced platelet count alone cannot explain the flow aggregation phenotype in these mice. Blood was drawn from 6 mice and images are representative of 10 fields of view per flow experiment. Scale bar: 10 μm.

Supplementary Figure 9

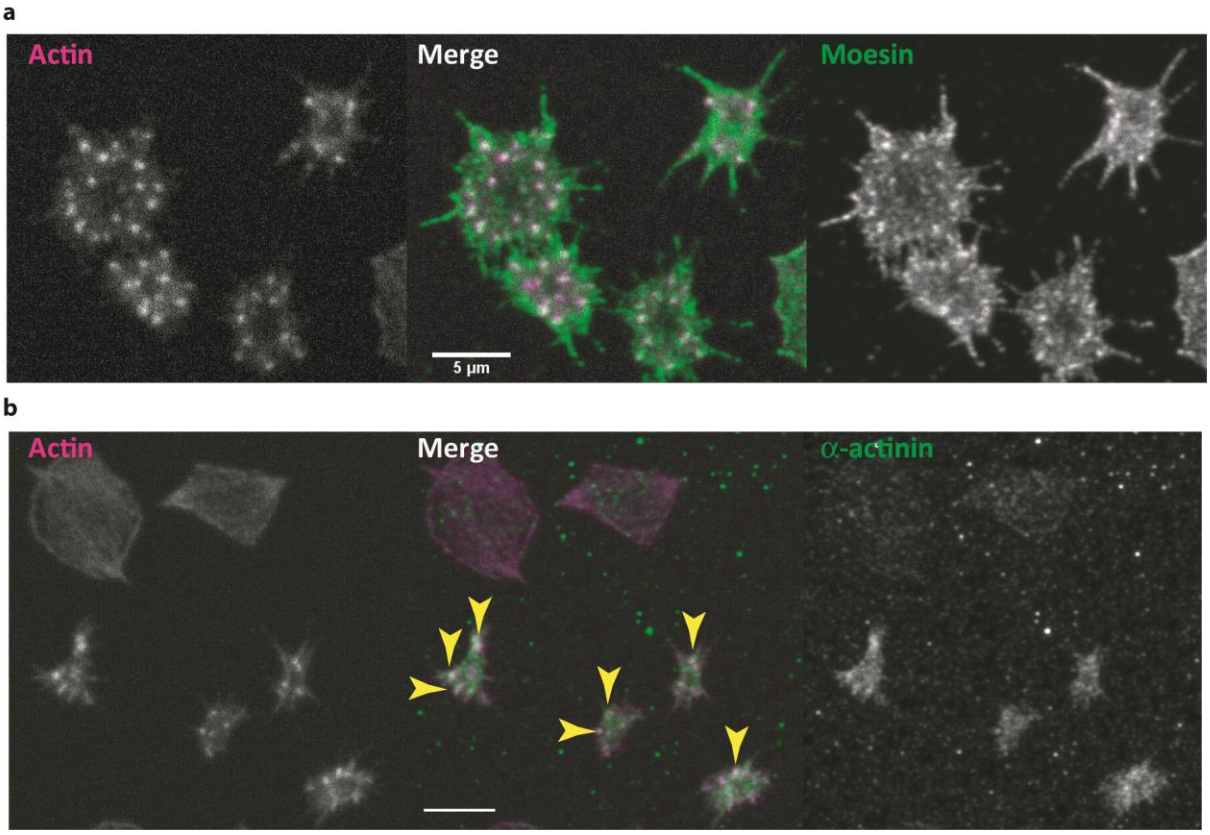

Moesin and α-actinin localisation in platelets.

Confocal images of human platelets spreading on fibrinogen and stained for F-actin (Alexa568-phalloidin, magenta) and either a) moesin (mouse anti-moesin, secondary anti-mouse Alexa-647; green) or b) α-actinin (rabbit anti-α-actinin, secondary anti rabbit Alexa-488, green). The merged image is shown in the middle panel. a) Moesin is present throughout the cell but is enriched at the actin nodule. b) Images show evidence that α-actinin is enriched at some actin nodules (arrowheads). Scale bar: 5 μm.

Supplementary Note 1

Actin nodules are not SICs

The enrichment of actin binding proteins at actin nodules and their presence at the very early stage of spreading indicates that they may be related to SICs which have been described in numerous cell types and share some proteins with podosomes and focal adhesions [1, 2]. Receptor for activated C kinase 1 (RACK1; also known as guanine nucleotide binding protein subunit b-like 1) is a marker for SICs and has been shown to play a role in integrin-mediated adhesion in Chinese hamster ovary fibroblast-like cells (CHO-K1 cells) [3]. Further, RACK1 is also expressed in platelets [4]. Immunolocalisation of RACK1 in human platelets spread on fibrinogen however shows no co-localisation of RACK1 with the actin nodules (Supplementary Fig. 3a). Additionally, nodules are devoid of RNA, another marker of these structures (Supplementary Fig. 3b), indicating that actin nodules are distinct from SICs.

Supplementary Note 2

Actin nodules do not degrade the fibrinogen matrix

The data presented in Figures 1 to 6 emphasise a number of similarities between actin nodules and podosomes in terms of their composition, protein localisation and dynamics. Actin nodules are considered too small to be podosomes, but the absence of classical podosomes in platelets and the number of similarities suggests that they are related actin structures. An important function of megakaryocyte podosomes is their ability to interact with and degrade the ECM via the action of matrix metalloproteinases (MMPs) [5]. This can be visualised as the appearance of matrix degradation in a fluorescently labelled ECM substrate at the location of podosomes [6]. We therefore investigated whether platelet actin nodules were able to degrade the ECM by assessing their ability to degrade fluorescent fibrinogen. We did not observe degradation of fluorescent fibrinogen in platelets with nodules or in fully spread platelets (Supplementary Fig. 5a). Furthermore, the broad spectrum MMP inhibitor GM6001 had no effect on platelet adhesion, spreading and nodule formation. Thus, there was no significant difference in the platelet number or surface area in 100 µM GM6001-treated versus vehicle-treated control platelets (Supplementary Fig. 5 b-d). There was also no significant difference in the number of actin nodules per μm^2 when MMPs were inhibited (Supplementary Fig. 5e), suggesting that MMPs do not play a role in actin nodule assembly and breakdown.

Supplementary References

[1] de Hoog, C.L., L.J. Foster, and Mann, M. RNA and RNA binding proteins participate in early stages of cell spreading through spreading initiation centers*. Cell*. **117**(5), 649-62. (2004).

[2] Cervero, P., et al., Proteomic analysis of podosome fractions from macrophages reveals similarities to spreading initiation centres*. Eur. J. Cell Biol.* **91**(11-12), 908-22. (2012).

[3] Cox, E.A., et al., RACK1 regulates integrin-mediated adhesion, protrusion, and chemotactic cell migration via its Src-binding site. *Mol. Biol. Cell*. **14**(2), 658-69. (2003).

[4] Senis, Y.A., et al., A comprehensive proteomics and genomics analysis reveals novel transmembrane proteins in human platelets and mouse megakaryocytes including G6b-B, a novel immunoreceptor tyrosine-based inhibitory motif protein. *Mol. Cell Proteomics*. **6**(3), 548-64. (2007).

[5] Schachtner, H., et al., Podosomes in adhesion, migration, mechanosensing and matrix remodeling. *Cytoskeleton (Hoboken)*, **70**(10), 572-89. (2013).

[6] Schachtner, H., et al., Megakaryocytes assemble podosomes that degrade matrix and protrude through basement membrane. *Blood*, **121**(13), 2542-52. (2013).
